# Role of hypoxia in Diffuse Large B-cell Lymphoma: Metabolic repression and selective translation of HK2 facilitates development of DLBCL

**DOI:** 10.1038/s41598-018-19182-8

**Published:** 2018-01-15

**Authors:** Kavita Bhalla, Sausan Jaber, Nanaji Nahid M., Karen Underwood, Afshin Beheshti, Ari Landon, Binny Bhandary, Paul Bastian, Andrew M. Evens, John Haley, Brian Polster, Ronald B. Gartenhaus

**Affiliations:** 1 Marlene and Stewart Greenebaum Comprehensive Cancer Center, University of Maryland, Department of Medicine, Baltimore, MD 21201 USA; 2 University of Maryland, Department of Biochemistry and Molecular Biology, Baltimore, MD 21201 USA; 3 Veterans Administration Medical Center, Baltimore, MD 21201 USA; 4 University of Maryland, Flow Cytometry Core, Greenebaum Comprehensive Cancer Center, Baltimore, MD 21201 USA; 5 Tufts Medical Center, Boston, MA 02111 USA; 6 Yale School of Medicine, Yale University, New Haven, CT 06520 USA; 7 National Institute on Aging, National Institutes of Health, Baltimore, MD 21224 USA; 8 Department of Pathology, Stony Brook Medicine, Stony Brook, NY 11794-8691 USA

## Abstract

Published molecular profiling studies in patients with lymphoma suggested the influence of hypoxia inducible factor-1 alpha (HIF1α) targets in prognosis of DLBCL. Yet, the role of hypoxia in hematological malignancies remains unclear. We observed that activation of *HIF1α* resulted in global translation repression during hypoxic stress in DLBCL. Protein translation efficiency as measured using ^35^S-labeled methionine incorporation revealed a ≥50% reduction in translation upon activation of HIF1α. Importantly, translation was not completely inhibited and expression of clinically correlated hypoxia targets such as *GLUT1*, *HK2*, and *CYT-C* was found to be refractory to translational repression under hypoxia in DLBCL cells. Notably, hypoxic induction of these genes was not observed in normal primary B-cells. Translational repression was coupled with a decrease in mitochondrial function. Screening of primary DLBCL patient samples revealed that expression of *HK2*, which encodes for the enzyme hexokinase 2, was significantly correlated with DLBCL phenotype. Genetic knockdown studies demonstrated that *HK2* is required for promoting growth of DLBCL under hypoxic stress. Altogether, our findings provide strong support for the direct contribution of *HK2* in B-cell lymphoma development and suggest that *HK2* is a key metabolic driver of the DLBCL phenotype.

## Introduction

Lymphoma is the fifth most common cancer^1,2^, with the most common subtype worldwide being diffuse large B-cell lymphoma (DLBCL) representing approximately 30–35% of all non-Hodgkin lymphomas (NHL)^3^. Rituximab plus cyclophosphamide, doxorubicin, vincristine and prednisone (R-CHOP) is a standard treatment for patients with DLBCL. However, only 50–60% of patients achieve long-term cure by this treatment. Further, cured patients are at increased risk of late effects (e.g., cardiac) disease due in part to chemotherapy. Additionally, it remains challenging to effectively treat refractory/relapsed cases with current regimens due in part to the intrinsic tumor heterogeneity of DLBCL^4^. Based on cell of origin (COO), DLBCL is most often classified as germinal center B cells (GCB) or activated B cell like (ABC). Based on transcriptional profiling, DLBCL can be classified as OxPhos-DLBCL or B cell receptor (BCR) DLBCL subtypes^5^. To add to complexity of phenotype, DLBCL patients fall into different prognostic categories; patients with GCB molecular signature have improved overall survival (60–70%) compared with that of patients with ABC signature (30–40%)^6,7^.

Lymphoid organs exhibit lower oxygen tension than blood and are hypoxic in nature^8^. B cells reside in bone marrow, which is hypoxic (pO_2_ 1.3%) with extravascular oxygen tension ranging between pO_2_ 0.6–2.8%. B-cells are localized in secondary lymphoid tissue like spleen where also they encounter hypoxic environment (pO_2_ 0.5–4.5%). Therefore, B-cells in the body are exposed to varying oxygen tensions^9^. Gene expression profiling studies highlighted the importance of tumor microenvironment and HIF1α targets in predicting DLBCL patients’ response to CHOP chemotherapy^10–14^. Despite the importance of hypoxic tumor microenvironment in B-cell development, not much is known about the role of hypoxia in hematologic malignancies, including DLBCL.

Hypoxia is a critical component of the tumor microenvironment and has profound effect on various metabolic pathways. Cells rapidly respond to hypoxic stress by undergoing translational arrest. Global translation is generally inhibited in various cell types under hypoxic stress^15,16^. This allows cells to spare energy for other important processes required to maintain cellular homeostasis. Therefore, as an adaptation to hypoxia, the ATP demand for protein synthesis rapidly drops to ~7% to protect cell from energy deprivation^17–20^. Repression in the rate of protein synthesis under hypoxia is dependent on two kinases, *PERK* and *mTORC*^15,21^. During hypoxia, cap-mediated translation is repressed by *mTORC*-dependent sequestration of eIF4E^22,23^. Recent studies demonstrated that *HIF1α* promotes hypoxia-induced cap dependent translation of selective mRNAs by up-regulation of translation initiation factor *eIF4E1* in breast cancer cells^24^.

Metabolism of tumor cell is reprogrammed for maximum consumption of glucose to provide carbon source for generation of nucleotides, amino acids and lipids required to drive tumor growth^25^. It was previously shown that in contrast to global repression, 33 key mRNAs were found to be resistant to repression including glucose transporters^15^. Initial step to metabolize glucose is catalyzed by an enzyme hexokinase II (HK2), which is highly expressed in aggressive tumors^26^. HK2 phosphorylates glucose to glucose-6- phosphate (G6P), which is then utilized by cells via major pathways of glucose metabolism including glycolysis, pentose phosphate pathway and glycogenesis to meet metabolic demand of a growing tumor^27^. Many malignancies of B-cell origin show increased glucose uptake and increase in lipid metabolism^6,28^. Expression profiling of genes involved in lipogenic pathway in B-cell aggressive lymphoma cases showed that adipophilin was highly expressed in patients of Burkitt lymphoma (BL), suggesting that adipophilin can be used as a metabolic target for diagnosis of BL^29^. During hypoxic stress, mitochondria function as an oxygen sensor to regulate cellular energy, reactive oxygen species, and cell death^30,31^. Mitochondria consume greatest amount of oxygen (80 to 95%) to allow oxidative phosphorylation (OxPhos), a primary metabolic pathway for ATP production^32^. Understanding regulation of various metabolic pathways in lymphoma may be beneficial to effectively target tumor microenvironment and improve therapeutic efficacy in DLBCL.

Our data delineate that hypoxic stress results in global repression of protein translation with selective stimulation of clinically correlated hypoxia targets such as *GLUT1*, *HK2* and *CTY-C*. This regulation in protein translation machinery is coupled with reduction in mitochondrial function and resistance of cell growth under hypoxic stress. Screening of human primary DLBCL patient samples determined that *HK2* is significantly correlated with lymphoma phenotype. Furthermore, we provide a proof of concept that *HK2* is involved in progression of DLBCL. Given the prognostic significance of *HIF1α* targets in predicting survival of DLBCL patients after treatment, the role of *HK2* in pathogenesis of hematological malignancies has not been explored before.

## Results

### *HIF1α* induction results in translation repression in DLBCL under hypoxic stress

**Figure 1:**
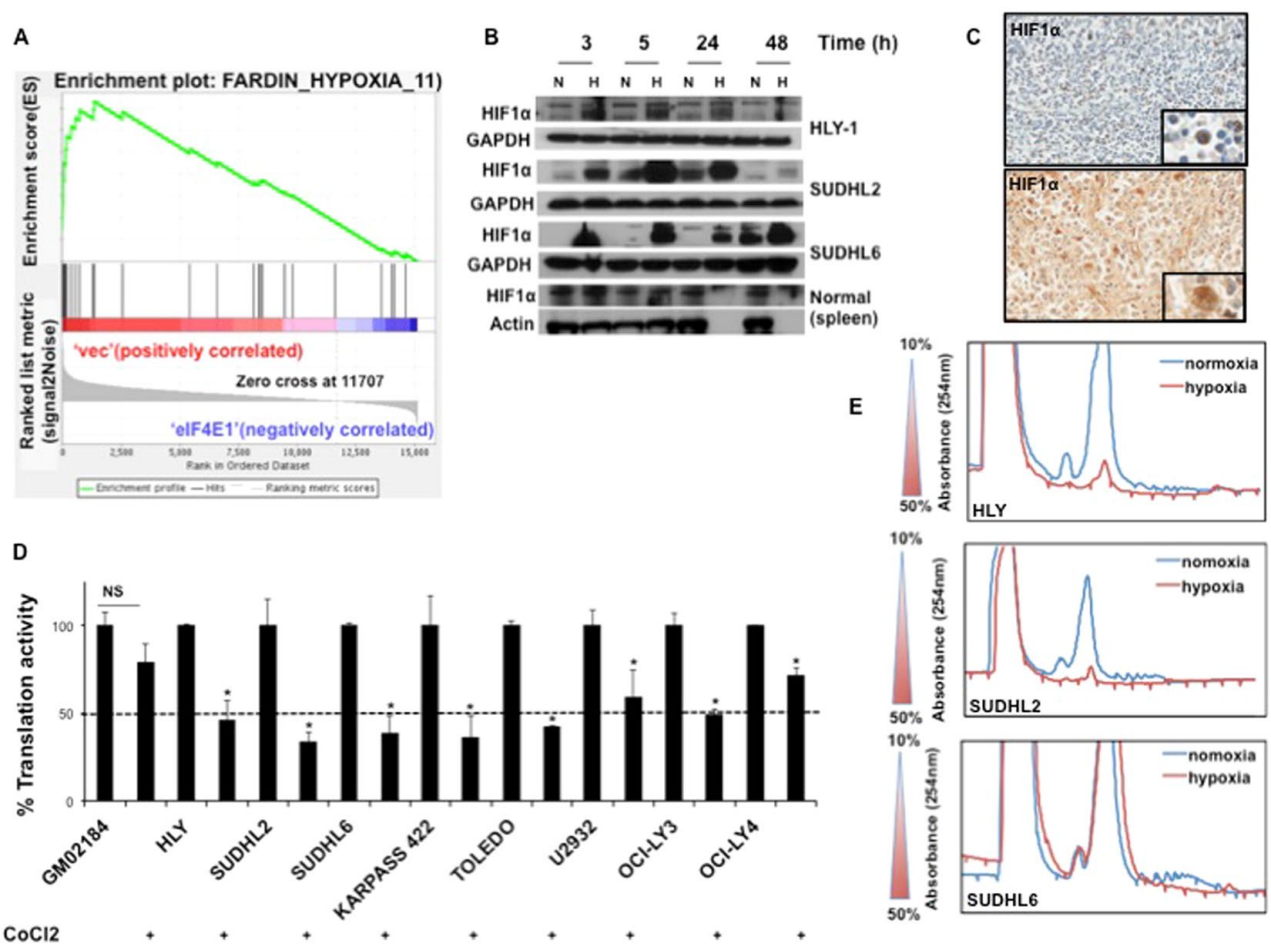
Hypoxia inhibits translation in DLBCL cells. (**A**) GSEA analysis of published eIF4E1 translatome to predict regulation of hypoxia targets in DLBCL (**B**). Activation of HIF1α in DLBCL cell lines. Western blot analysis to determine HIF1α expression in hypoxia following 3, 5, 24 or 48 h of hypoxia. (**C**) IHC of HIF1α in malignant DLBCL and normal lymphoid tissue (**D**) Global repression in translation under hypoxia Cells were treated with CoCl_2_ for 48 h. This was followed by labeling with (^35^S-methionine) for an hour. Translation activity was measured by calculating the amount of ^35^S-met incorporated into protein by using a scintillation counter. Translation efficiency was set to 100% for cells cultured under normoxia. Data is plotted as an average of n = 3 ± SE and was normalized to the cell number. (**E**) Polysomal profiling is depicted for DLBCL cells HLY, SUDHL2 and SUDHL6 cultured under normoxia or hypoxia for 48 h. Sucrose gradient ranging from 10 to 50% is displayed on the left. Examples of changes in polysomal profiles in different DLBCL cell lines cultured under normoxia and hypoxia are shown on the right. Blue line represents control and red represents reduced mRNA translation under hypoxia.

eIF4E1 is one of the best characterized subunits of translation initiation factor complex eIF4F, which plays an important role in malignant transformation^33^. We utilized our published translatome data-set from HLY DLBCL cells overexpressing *eIF4E1*^34^ to analyze a clinically relevant Faradin hypoxia gene signature^35^ in order to examine the relationship between hypoxia and translation in DLBCL. GSEA analysis showed that hypoxia signature was enriched in vector control dataset, which was significant by both FDR q value (2.4 E-4) and FWER p value, 0.003. However, *eIF4E1*-overexpressing dataset was inversely correlated to hypoxia signature suggesting that protein translation might be compromised during hypoxic stress (Fig. 1A). One of the important responses to hypoxia is repression of global mRNA translation^18,21,36,37^. Cells undergo this adaptive response to maintain energy homeostasis. We first determined if the level of HIF1α was altered under hypoxic stress in DLBCL cell lines HLY, SUDHL2 and SUDHL6. Cells were grown under normoxia and 1% hypoxia for 3, 4, 24 and 48-hour time points to determine *HIF1α* levels in respective cell lines. Induction of *HIF1α* protein was observed at almost all time points under hypoxic stress in DLBCL cells compared with cells that were cultured at 21% oxygen (Fig. 1B). However, in normal primary B-cells, only a short-term induction of *HIF1α* was observed for 3 hours with no change at 5 hours. Furthermore, normal primary B-cells did not survive prolonged hypoxic stress when cells were exposed to 1% hypoxia for 24 to 48 hours (Fig. 1B). Expression of HIF1α was also induced in malignant lymphoid tissue obtained from a DLBCL patient sample compared to that of a normal lymphoid tissue (Fig. 1C). Next, we determined the effect of *HIF1α* induction on protein translation in several DLBCL cell lines. Protein translation was reduced by 50% or greater upon activation of *HIF1α* (Fig. 1D). No significant inhibition in translation was observed in the non-malignant B-cell line GM02184. Polysomal profiling also confirmed translation repression in DLBCL cells under hypoxia (Fig. 1E). Analysis of the phosphorylation status of mTOR targets ribosomal protein S6 (RPS6) and S6 kinase (p70S6K) demonstrated that mTOR signaling might be partly responsible for translation repression in SUDHL2, but not in HLY in presence of hypoxia (Fig. S1). These results indicate that translation of HIF1α is refractory to hypoxic stress. Thus, it is likely that expression of HIF1α target genes that are required to adapt to hypoxic stress is resistant to translational inhibition in DLBCL cell lines.

### Enrichment of clinically correlated HIF1α targets in DLBCL

To understand the global transcriptome profile regulated in DLBCL, we performed microarray analysis using Illumina bead microarray. Data generated was subjected to co-correlation analysis to predict RNA enrichment of clinically relevant hypoxia targets in our dataset. The notable genes that were found to be up-regulated in HLY and SUDHL2 included members of glucose and lipid metabolism (*SLC2A1*, *PDK1, HK2, PLIN2, INSIG2, ZNF395, GLRX, SCRAB1, VEGFA, PKFB3, NDRG1, CXCR4 and PLIN2*), cell cycle growth regulation (*CCNG2, GADD45B, CAV1 and CCND3*), protein tyrosine kinases (*MET and FYN*), cytochrome family (CYB5A), G-protein coupled receptor superfamily (*ADORA2B, RRAGD, OSTM1 and LSG1*), serine threonine kinases (*PIM1 and PRKCA*), zinc finger protein family (*BCOR, ZMYND8, and EGR1*), DNA repair pathway (*REDD-1*) and genes involved in calcium regulation (*DPYSL2, ITPR1, S100A4 and S100A6*) (Fig. S2A and B). The gene expression program that distinguishes SUDHL2 from HLY includes markers of ribosomal protein (RRP12), heat shock proteins (*HSPH1, HSPA4*) isocitrate dehydrogenase (*IDH3a*), solute carrier family 6 protein (SLC5A6), gene encoding for RNA polymerase III (*POLR3K*) and RNA binding protein (*RPP25*) (Fig. S2B). Initial data analysis of SUDHL2 dataset failed to detect *GLUT1* and *HK2* because of the highly significant pre-established criteria set for RNA enrichment co-correlation analysis. However, when raw data was analyzed for glucose transporters in SUDHL2 dataset, we observed a four-fold induction of *GLUT1* and 1.8 fold induction of *HK2* under hypoxia (Fig. S2C).

Ribosomal proteins are highly sensitive to translational repression^15^; we therefore determined expression of ribosomal genes regulated in our data set. In HLY, some of the important up-regulated ribosomal genes include *RPL28, RPL36A and RPS23*; significantly down-regulated ribosomal genes identified were *RPL26L1, MRPL41, MRPL43, and MRPS2*. In SUDHL2, up-regulated ribosomal genes include *MRPL41 and RPL26L1*; ribosomal genes with down-regulated expression included *RPS23*, *RPL36A*, *RPS27L, RPL28, RPL15, RPL22* and *RPF2*. It appears that ribosomal targets are differentially regulated in two datasets, HLY and SUDHL2 (Fig. S2D).

Lymphoma cells are characterized as oxidative tumors, indicating the requirement of mitochondrial function for tumor progression^38^. We also explored regulation in HIF1α-stimulated mitochondrial pathways by Ingenuity Pathway Analysis (IPA) of the microarray data. The most significantly regulated pathways were related to mitochondrial dysfunction and mitochondrial membrane potential in HLY (Fig. S3A) and SUDHL2 (Fig. S3C). Detailed lists of genes are provided in Fig. S2B and D. Taken together, our dataset pointed out that gene expression profiles that were similarly regulated by *HIF1α* in both HLY and SUDHL2-DLBCL cells were the expression of glucose and mitochondrial metabolism genes. These genes are of particular importance as they are required for cell survival during oxygen limitation and may perhaps influence malignant DLBCL phenotype.

### Selective activation of metabolic genes under hypoxia

**Figure 2:**
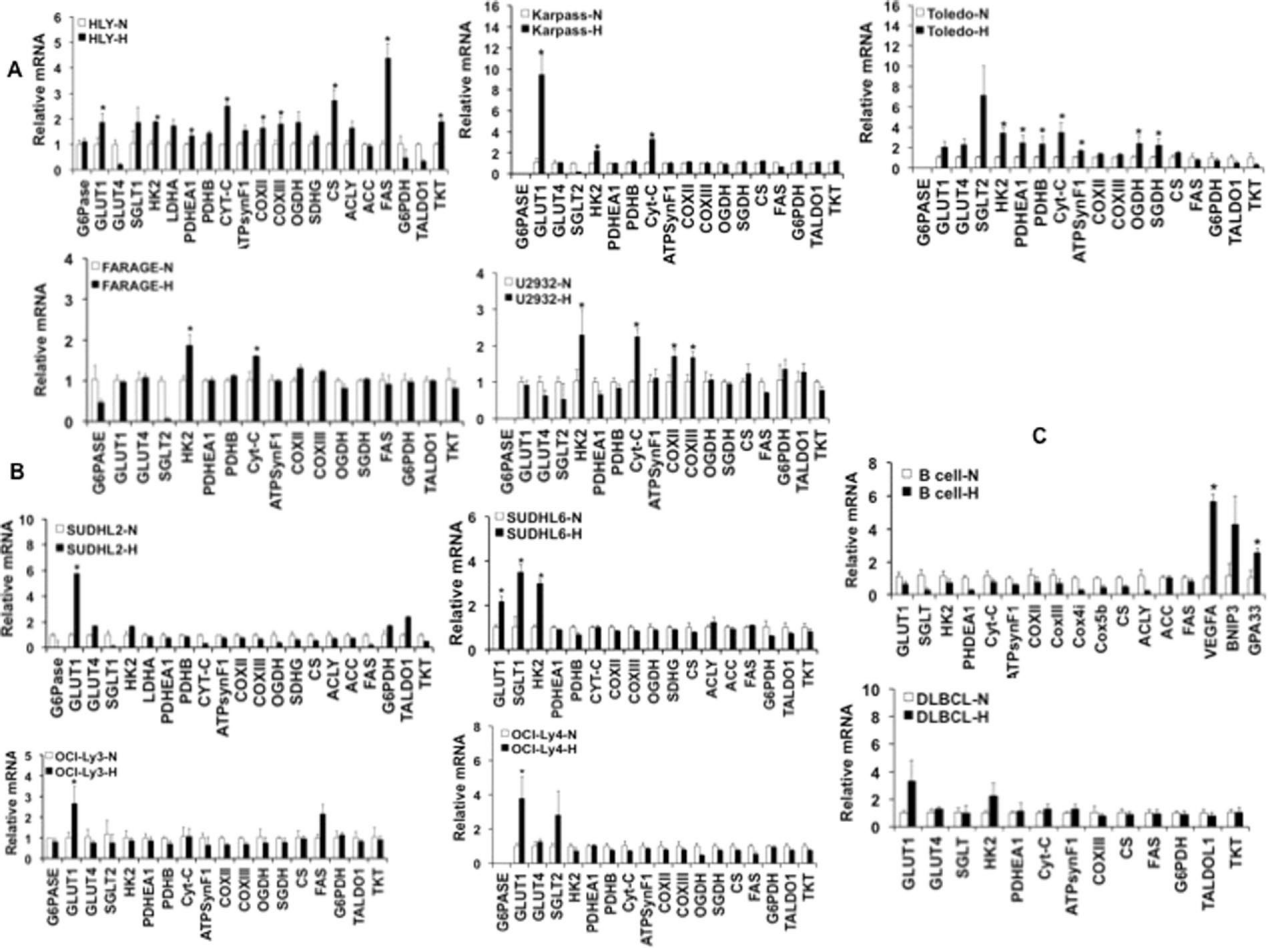
Expression of metabolic targets in DLBCL. RNA expression analysis of metabolic genes involved in glucose metabolism (*G6pase, GLUT1, GLUT4*, *SGLT1*, *HK2, LDHA, PDHEA1, PDHB*); oxidative phosphorylation (*Cyt-C, ATPsynthase F, COXII, COXIII, OGDG, SGDH*); Lipid metabolism (*CS, ACLY, ACC, FAS*); PPP (*G6PDH, TALDO1,TKT*) in DLBCL cell lines and primary B cells obtained from a malignant diffuse B cell lymphoma patient or from a normal spleen tissue. (**A**) DLBCL cell lines found to exhibit an OxPhos phenotype along with increase in glucose metabolism genes (**B**) DLBCL cell lines with an increase in glucose transporters and a non-OxPhos phenotype. RNA expression in primary B cells is shown in (**C**) (top panel, normal B cell and bottom panel, diffuse large B cell). The expression of respective genes was normalized to GAPDH expression. Values are presented as n = 3 ± SD. Asterisks define significant differences p < 0.05.

Expression of genes involved in regulation of glucose and mitochondrial metabolism as highlighted by microarray data was validated by RT-PCR using various diffuse large B-cell lymphoma cell lines and primary B cells derived from lymphoma patients and normal lymphoid tissues. Rapid response in transcriptional changes was assessed after exposure of DLBCL cells to 1% hypoxia for 3–4 hours. Examination of gene expression profiles in various DLBCL cell lines revealed differences in their dependency on metabolic pathways. For example HLY, Karpass 422, Toledo, Farage, and U2932 cell lines showed increase in OxPhos phenotype (Fig. 2A). Expression of *Cytochrome-C* was also significantly stimulated in all cell lines with the OxPhos subtype. OxPhos subtype cells also showed a significant increase in expression of mitochondrial membrane-associated gene *HK2*. However, certain cell lines such as SUDHL2, SUDHL6, OCI-Ly3 and OCILY4 did not show an increase in OxPhos markers, not even the critical mitochondrial protein cytochrome C, which was elevated in the other cell lines (Fig. 2B). This group of cell lines had increase in expression of glucose transporters *GLUT1*, *SGLT2* and *HK2*. SUDHL2 cells were also found to have increased expression of pentose phosphate pathway (PPP) genes indicating the possibility of nucleotide precursors being synthesized by these cells under hypoxia. Interestingly in the HLY cell line, we also observed increased expression of genes involved in fatty acid synthesis (*FASN*). In addition *SLC25A1* (*CS*), which is required for transport of citrate from mitochondria to cytoplasm was induced under hypoxic stress. Another marker, *ACLY*, that is involved in the conversion of citrate into acetyl coenzyme A for fatty acid synthesis was also increased in expression in hypoxic samples compared to normoxic controls (Fig. 2A). Although, we noticed an increasing trend in expression of FAS in OCILy3, it was not significantly different under hypoxia. This suggests that increased OxPhos might be coupled with increased fatty acid synthesis in certain DLBCL cell lines like HLY.

Malignant primary B-cells obtained from DLBCL patients exhibited a transcriptional profile similar to non-OxPhos phenotype (Fig. 2C), as observed in cell lines depicted in Fig. 2B. Importantly, we did not see an increase in expression of glucose transporters and mitochondrial markers in B cells derived from normal spleen tissue (Fig. 2C). RNA quality of normal primary B-cells was confirmed by determining activation of other HIF1α targets such as VEGFA, BNIP3 and GPA33 as controls for the experiment (Figs 2C and S4). We also noticed that expression of enzymes not essential for cell function, for example gluconeogenic enzyme, *G6pase*, was either not changed or was inhibited in the hypoxic state. Hence, our transcriptional profiling data depicts that DLBCL cells engage both a glycolytic and OxPhos-dependent metabolism, confirming the microarray data. These RNA expression studies indicate that DLBCL cell lines converge to a common mechanism with increased expression of genes involved in glucose metabolism suggesting the importance of these markers in manifestation of the DLBCL phenotype.

### Effect of hypoxic stress on mitochondrial function in DLBCL

**Figure 3:**
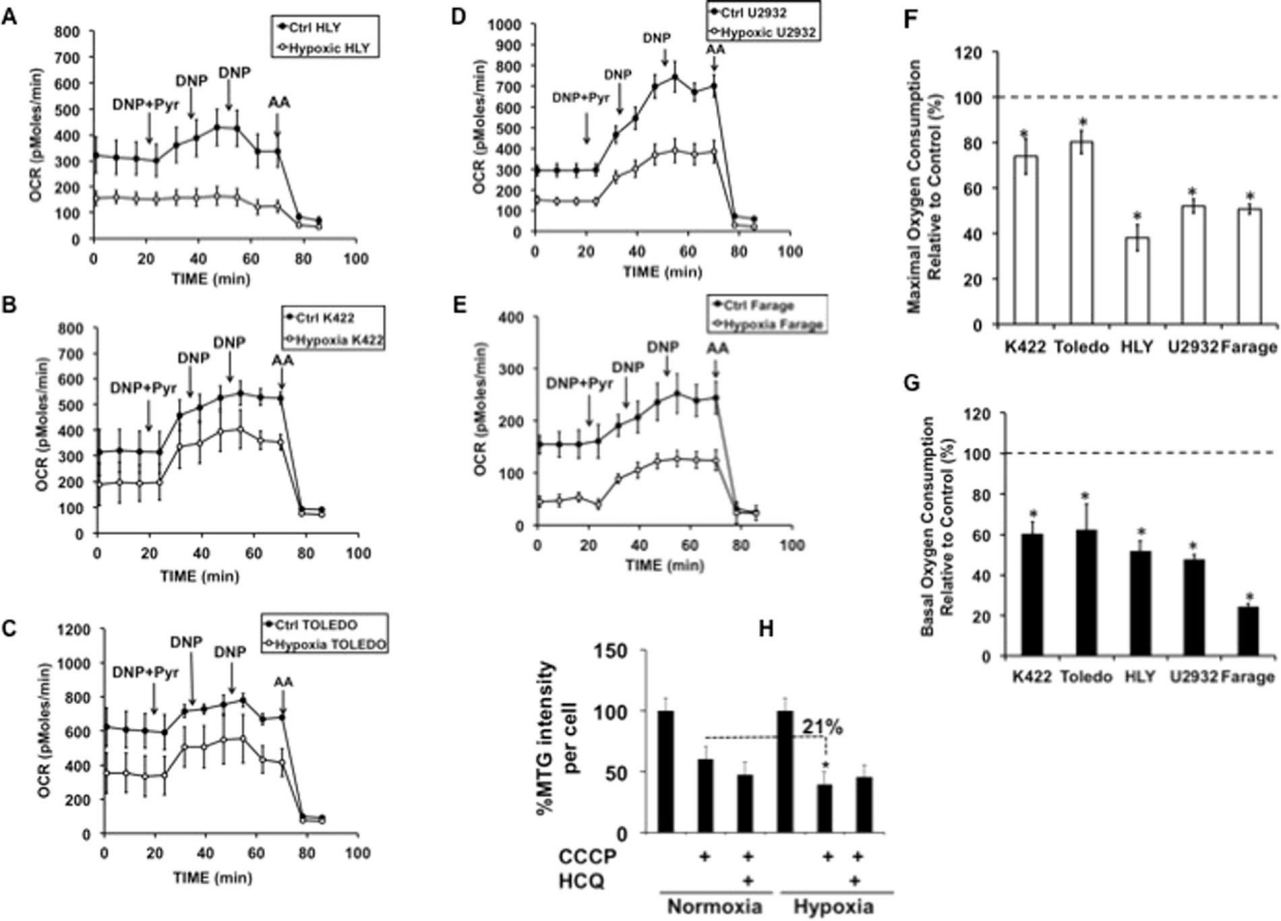
Regulation of mitochondrial function under hypoxic stress in DLBCL. (**A–E**) Representative oxygen consumption rate (OCR) traces from OxPhos subtype DLBCL cells. Cells were exposed to normoxia and hypoxia overnight and the next day mitochondrial respiration was measured during the sequential addition of uncoupler DNP plus 10 mM pyruvate, DNP alone twice, and finally an inhibitor of respiration antimycin A. Data are derived from n = 3 passages per cell line. (**F** and **G**) Maximal and basal respiration rates under hypoxia compared to control (cells maintained in normoxia). For maximal respiration *p values obtained for DLBCL cell lines were p < 0.028 for K422, p < 0.017 for Toledo, p < 0.001 for HLY, U2932 and Farage respectively. For basal respiration *p values obtained were p < 0.003 for K422, p < 0.040 for Toledo, p < 0.001 for HLY, U2932 and Farage respectively. (**H**) Cells were treated with CCCP overnight followed by treatment for 3 h before analysis with HCQ (to block lysosomal degradation). Flow cytometry was used to determine Mito Tracker green (MTG) staining, p < 0.0003 for difference in MTG intensity compared under normal and hypoxic culture conditions.

It is well known now that mitochondrial function is not always disrupted in cancer cells as earlier proposed by Otto Warburg^39^. In our expression data, cytochrome-c was induced in HLY, K-422, Toledo, U2932, and Farage cell lines. It is an important oxygen sensor and a critical regulator of OxPhos^40^. It was shown earlier that under normoxic culture conditions OxPhos subtype DLBCL cell lines displayed an increase in the rate of oxygen consumption using palmitate as a substrate^7^. This led us to hypothesize that mitochondrial bioenergetics function is enhanced in the OxPhos subtype of DLBCL cells under hypoxia. We first measured mitochondrial respiration in HLY, K-422, Toledo, U2932, and Farage cells. Changes in maximal respiration of DLBCL cells under hypoxia was determined by sequential addition of DNP^41^ and antimycin A. Oxygen consumption rate OCR was significantly reduced when HLY, K-422, Toledo, U2932 and, Farage cells were maintained in hypoxia compared to the normoxic control condition (Fig. 3A–E). Quantitation of this data showed a significant decrease in basal respiration rate of 40 to 60% and maximal respiration was dropped by 25–50% in the hypoxic cells compared to normoxic controls. The respiration rate for cells grown under regular culture conditions were set to 100% (Fig. 3F,G). Compared to normoxic control, hypoxic cell lines showed unchanged ATP synthesis-dependent OCR, suggesting that cells were not energy deprived (Fig. S5A).

To determine if there is a decrease in mitochondrial mass that may be contributing to the decrease in OCR during hypoxic stress, we treated HLY cells with a low dose of CCCP, an inducer of mitophagy and HCQ, an inhibitor of lysosomal degradation^42,43^. Mitochondrial population levels were estimated using MitoTracker Green. Hypoxia treatment induced a 21% decrease of MitoTracker green fluorescence in HLY samples treated with CCCP compared to normoxic HLY cells treated (Figs 3H and S5B). No change in MitoTracker fluorescence was detected in cells treated with HCQ under normoxia and hypoxia suggesting that the apparent decrease in mitochondrial mass during hypoxia is not a result of lysosomal degradation. We then determined if the decrease in OCR was associated with changes in mitochondrial cytochrome-c expression. Microscopic examination of hypoxic cells depicted a decrease in mitochondrial cytochrome-c expression relative to TOM20 and cytochrome-c expression (Fig. S5C,D). Our mitochondrial data showed that the influence of hypoxia is not consistent in mitochondrial functional effects and nuclear/cytoplasmic OxPhos expression. Despite an increase in expression of nuclear/cytoplasmic OxPhos we observed suppression in mitochondrial oxygen consumption and mitochondrial cytochrome-c expression in DLBCL cell lines during hypoxic stress.

### Effect of hypoxia on cell growth and cell cycle regulators *in vitro*

**Figure 4:**
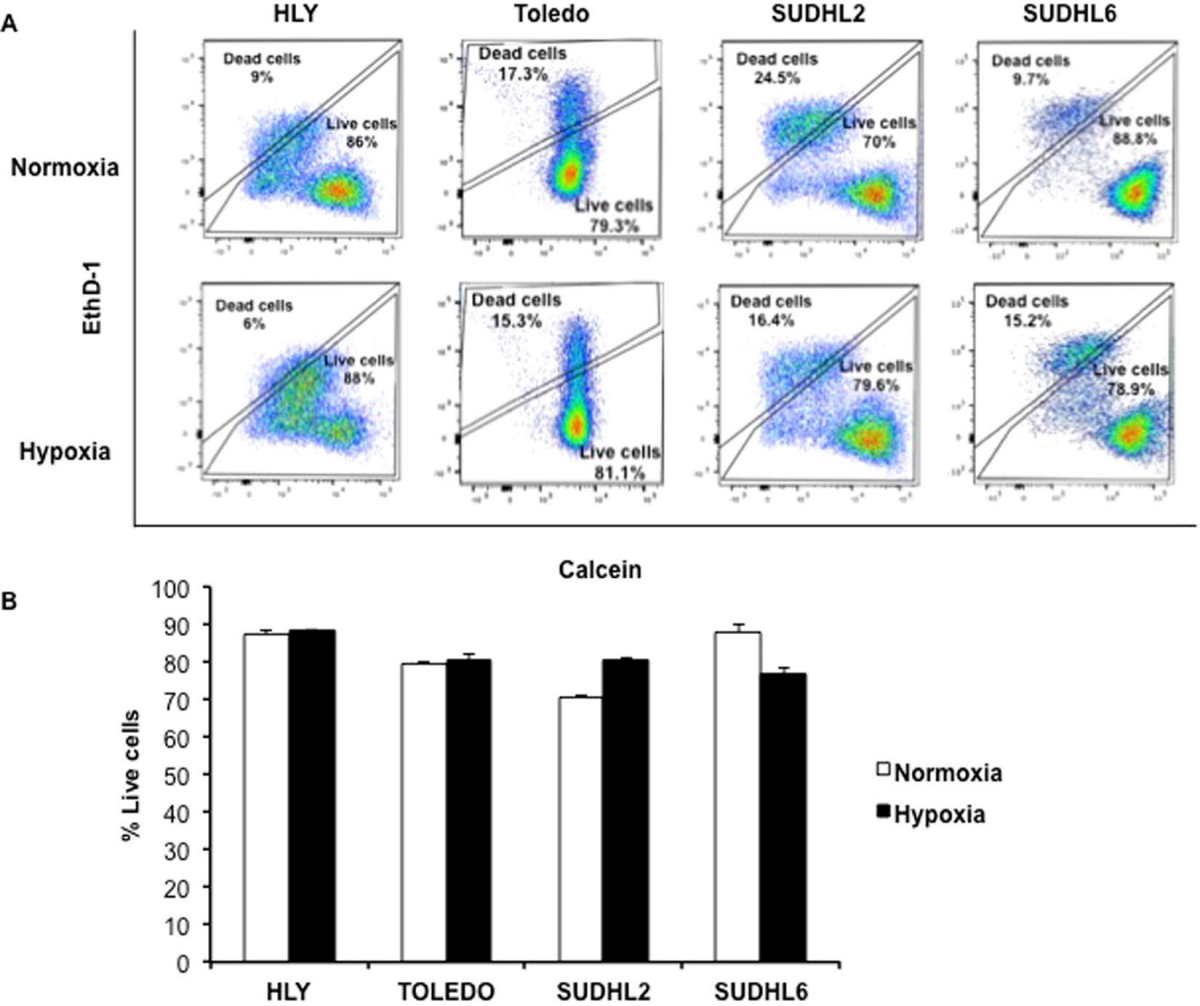
Cell growth of lymphoma cell lines under hypoxia. (**A**) Quantitation of live and dead cells in lymphoma cells after incubation in 1% oxygen (hypoxia) for 24 hours using Calcein- AM (Calcein acetoxymethyl ester) and EthD-1 by flow cytometry (FACS CANTO II), n = 3 ± SD. Representative images of live and dead cells in DLBCL cell lines HLY, SUDHL2 and SUDHL6 as determined by flow cytometry are depicted. (**B**) Results are expressed as the percentage of live cells in DLBCL cell lines under normoxia and hypoxia, respectively.

Studies have shown that regulation of translational machinery and mitochondrial oxygen consumption has a profound effect on cell growth. To determine the effect of hypoxic stress on survival of DLBCL cell lines, we measured cell proliferation and cell cycle distribution in HLY and TOLEDO (OxPhos ABC subtype), SUDHL2 (non-OxPhos ABC subtype), and SUDHL6 (non-OxPhos GC subtype). We first performed live and dead assay to determine cell growth under hypoxic stress. The viability of DLBCL cells was assessed by flow cytometry with calcein-AM and nucleic acid dye EthD-1 for direct observation of live and dead cells, respectively. Representative dot blot flow data are shown in Fig. 4A for DLBCL cell lines HLY, Toledo, SUDHL2 and SUDHL6. As reflected by flow quantification of live and dead cell staining, DLBCL cell lines demonstrated less cell death after hypoxic exposure for 24 hours compared to cells maintained at normal culture conditions. In SUDHL6 we observed 6% more cell death under hypoxic stress. Importantly, live cells count suggested that there is a cessation in growth of DLBCL cell lines under hypoxic stress, which is a classic response to hypoxia (Fig. 4B). To examine a possibility of hypoxia-induced growth arrest in DLBCL cell lines, we subjected these cells to hypoxia for 24hrs and performed PI staining. We observed a 1.8 fold increase in percentage of cells arrested in G2/M phase in SUDHL2 and 1.6 fold increase in SUDHL6, respectively (Fig. S6A). No significant change in G2/M arrest was observed in DLBCL cell lines HLY and Toledo. We also explored expression of genes known to regulate G2/M cell cycle arrest in our microarray datasets for HLY and SUDHL2. Members of the phosphatase family *CDC25* *A*, *CDC25B* and *CDC25C* dephosphorylate cyclin-dependent kinases to regulate cell cycle. *CDC25* *A* and *CDC25* *C* are involved in G1/S phase transition. *CDC25B* is involved in G2/M transition and mitotic induction. It dephosphorylates *CDC2* and results in its activation to regulate mitotic induction in cell cycle^44,45^. G2/M arrest in SUDHL2 was accompanied by a decrease in levels of *CDC25B* phosphatase and its target *CDC2* (Fig. S6B), whereas an increase in expression of these phosphatases and *CDC2* was observed in HLY (Fig. S6C). Enrichment of *CDC25A* and *CDC25C* was not observed in the SUDHL2 dataset. Together, *in vitro* growth data suggest that growth of lymphoma cell lines HLY, Toledo, SUDHL2 and SUDHL6 were resistant to hypoxic stress.

### *GLUT1*, *CYT-C* and *HK2* expression in human DLBCL patient samples

**Figure 5:**
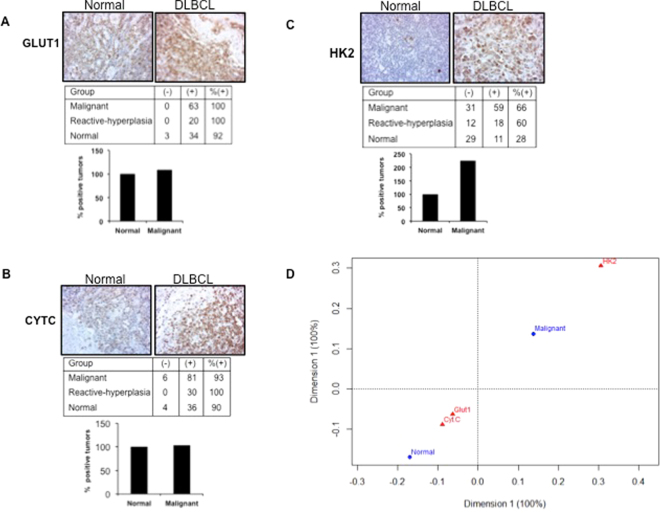
Tumor tissue microarray staining and analysis of DLBCL patient samples. Summary of lymphoma tissue microarray expression of *GLUT1*, *Cyt-C* and *HK2* staining are depicted in respective tables (**A**), (**B**) and (**C**). Number of tumor cores negative for staining is shown in the column marked as (−) and tumor cores with positive staining are depicted in the column marked as (+). Positive percentage tumor for each staining is indicated. The percentage difference in the number of positive tumors in the normal and malignant group for expression of *GLUT1*, *Cyt-C* and *HK2* in shown as a bar graph with scale set to 100% for the normal group in each case. (**D**) Correspondence analysis of array expression data to establish the relationship between marker and phenotype. *HK2* was closely related to the malignant group compared to the other markers *GLUT1* and *Cyt-C*, which were closer to the normal group. The position of a variable on the CA graph from the origin indicates the extent of similarity of its response profile compared to the average. The markers *GLUT1* and *Cyt-C* are closer to the origin, which indicates that their contribution to the observed phenotype is small. The farther location of *HK2* implies a larger deviation from the expected contribution and its importance to the observed phenotype.

Gene expression studies identified three distinct genes *GLUT1*, *HK2* and *Cyt-C* that might play a critical role in predicting outcome for DLBCL. Increased *GLUT1* expression results in increased glucose transport. High levels of *HK2* are required for tumor initiation^46,47^. Cytochrome-c was found to be associated with drug resistance in thymic lymphoma cells^48^. We therefore performed screening of lymphoma tissue microarrays (Biomax) to validate the expression of metabolic markers *GLUT1*, *Cyt-C* and *HK2* in human DLBCL patient samples and explore their prognostic importance. Immuno-histochemical staining showed that expression of *GLUT1*, *Cyt-C* and *HK2* is induced in DLBCL samples compared to normal lymphoid tissue (Fig. 5A–C). The image for respective tissue disc on the tissue arrays was scored. Quantitation of tumor tissue microarray revealed that all 63 (100%) tumors were positive for *GLUT1* staining in the malignant group. Although there was increased expression of *GLUT1* in tumor cores, 92% of normal tissue cores also were positive for *GLUT1* expression. This difference in *GLUT1* expression was similar in the reactive hyperplasia group when compared to normal tissue *GLUT1* (Fig. 5A). The percentage of tumors positive for *Cyt-C* expression in the malignant group was 93% and in the reactive hyperplasia group was 100%, compared to 90% in the normal samples (Fig. 5B). Analysis of *HK2* expression revealed that 66% of tumors were positive for *HK2* expression and 60% of reactive hyperplasia tumors were positive for *HK2* expression. However, staining of discs derived from normal samples showed that very few normal samples, i.e. only 28%, were positive for *HK2* expression (Fig. 5C). We observed a greater than 2-fold increase in the number of DLBCL samples that were positive for *HK2* expression. Such a marked difference between the percentage of normal samples and DLBCL patient samples positive for HK2 expression was not noticed in case of *GLUT1* and *Cyt-C* expression. Interestingly, correspondence analysis indicates that *HK2* expression was close to malignant group, while, expressions of *GLUT1* and *Cyt-C* in DLBCL were close to that in normal samples (Fig. 5D). Therefore, compared with *GLUT1* and *Cyt-C*, *HK2* appears to be a critical metabolic driver of DLBCL phenotype. Our data clearly determine the clinical relevance of *HK2* in lymphoma progression.

### HK2 is selectively translated by eIF4E1 in DLBCL under hypoxic stress

**Figure 6:**
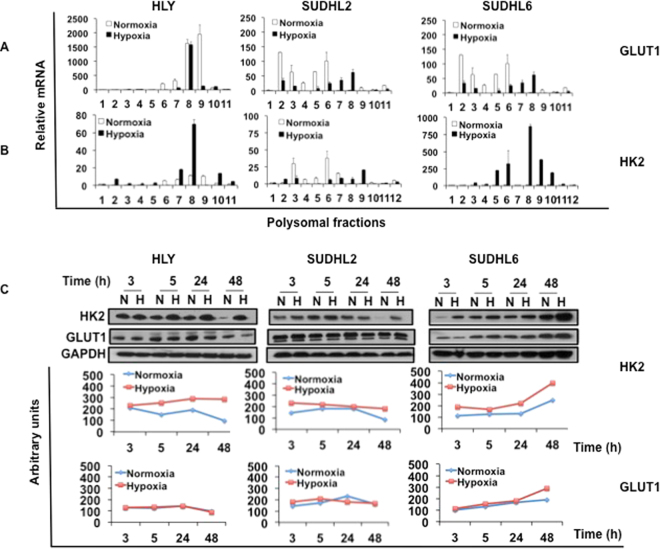
Effect of hypoxia on translation of *GLUT1* and *HK2* in DLBCL cell lines. Polysomal analysis of *GLUT1* and *HK2* in HLY, SUDHL2 and SUDHL6 cells maintained under normoxia or hypoxia. RNA was isolated from each polysomal fraction using Trizol and was subjected to RT-PCR analysis. Distribution of (**A**) *GLUT1* and (**B**) *HK2* in different polysomal fractions. Increased *GLUT1* and *HK2* levels were associated with heavier polysomal fractions ranging from 7–12. (**C**) DLBCL cells were exposed to normoxia and hypoxia for the indicated time points. The amount of *GLUT1* and *HK2* protein was detected by immunoblot analysis. *GAPDH* was used as a loading control. Protein expression for 3, 5, 24 and 48 h was quantitated using Image J software and is depicted in panels below the respective protein blots.

We further determined if glucose transporters *HK2* and *GLUT1* were efficiently translated under hypoxic stress. RT-PCR analysis of polysomal RNA depicted an increased association of *GLUT1* and *HK2* to heavier fractions of hypoxic polysomes in DLBCL cell lines after 24 hours of hypoxic exposure (Fig. 6A,B). Western blot analysis showed that *HK2* protein expression was induced at all time points in lymphoma cell lines exposed to hypoxia compared to those grown under normoxia. In contrast, *GLUT1* expression was not induced at all time points (Fig. 6C). This indicates that *HK2* is efficiently translated under hypoxic stress in DLBCL compared to *GLUT1*. Since the function of *eIF4E1* has been implicated in translation of genes associated with malignant transformation^49–51^, we next asked if eIF4E1 plays a role in translation of *HK2*. However, studies on the regulation of *eIF4E1* expression and its phosphorylation during hypoxia are conflicting. Hypoxia resulted in reduced expression of peIF4E1 at an early (3 hours) and late time (48 hours) points in HLY (Fig. S7A). To our surprise, hypoxia did not lead to a decrease in the overall abundance of eIF4E1 in HLY and SUDHL2 cell lines (Fig. S7A), despite the *eIF4E1*-expressing HLY dataset being negatively correlated to hypoxia targets. Wouters and colleagues^52^ also did not observe any change in expression of *eIF4E1* when HeLa cells were exposed to hypoxia for 0 to 16 hours. After 24 hours, its phosphorylation was slightly reduced, which is similar to what we observed in DLBCL cell lines HLY and SUDHL2. This suggests that hypoxia does not directly regulate levels of *eIF4E1*, but rather affects translation of proteins such as *HK2* that might compete for the availability of *eIF4E1* for its selective translation during hypoxic stress. To test this possibility, expression of *eIF4E1* and *HK2* were determined in polysomes isolated from HLY and SUDHL2. *eIF4E1* and *HK2* were enriched in polysomal fractions (Fig. S7B and C).

We also overexpressed *eIF4E1* in HLY cells to determine the effect of exogenous *eIF4E1* in the regulation of *HK2* protein levels. The presence of eIF4E1 significantly induced *HK2* expression in HLY cells when exposed to 1% hypoxia for 48 hours (Fig. S7D). Next we directly determined whether the presence of *eIF4E1* induces *HK2* luciferase promter activity together with *HIF1α* expression (Fig. S7E). Therefore, we transfected Cos7 cells with *HK2* promoter linked to a luciferase reporter in the presence and absence of *eIF4E1* and *HIF1α*. Our data show that while *eIF4E1* activates the *HK2* promoter, *HIF1α* strongly coactivates expression of *HK2*. These effects were further stimulated by hypoxic exposure of cells. *HK2* promoter activity increased by 2-fold in normoxia and about 4-fold in hypoxia in the presence of *eIF4E1* and *HIF1α*. Therefore *eIF4E1*, along with *HIF1α* activation, is responsible for the hypoxia-induced expression of *HK2*.

### *HK2* promotes DLBCL growth *in vitro* and *in vivo*

**Figure 7:**
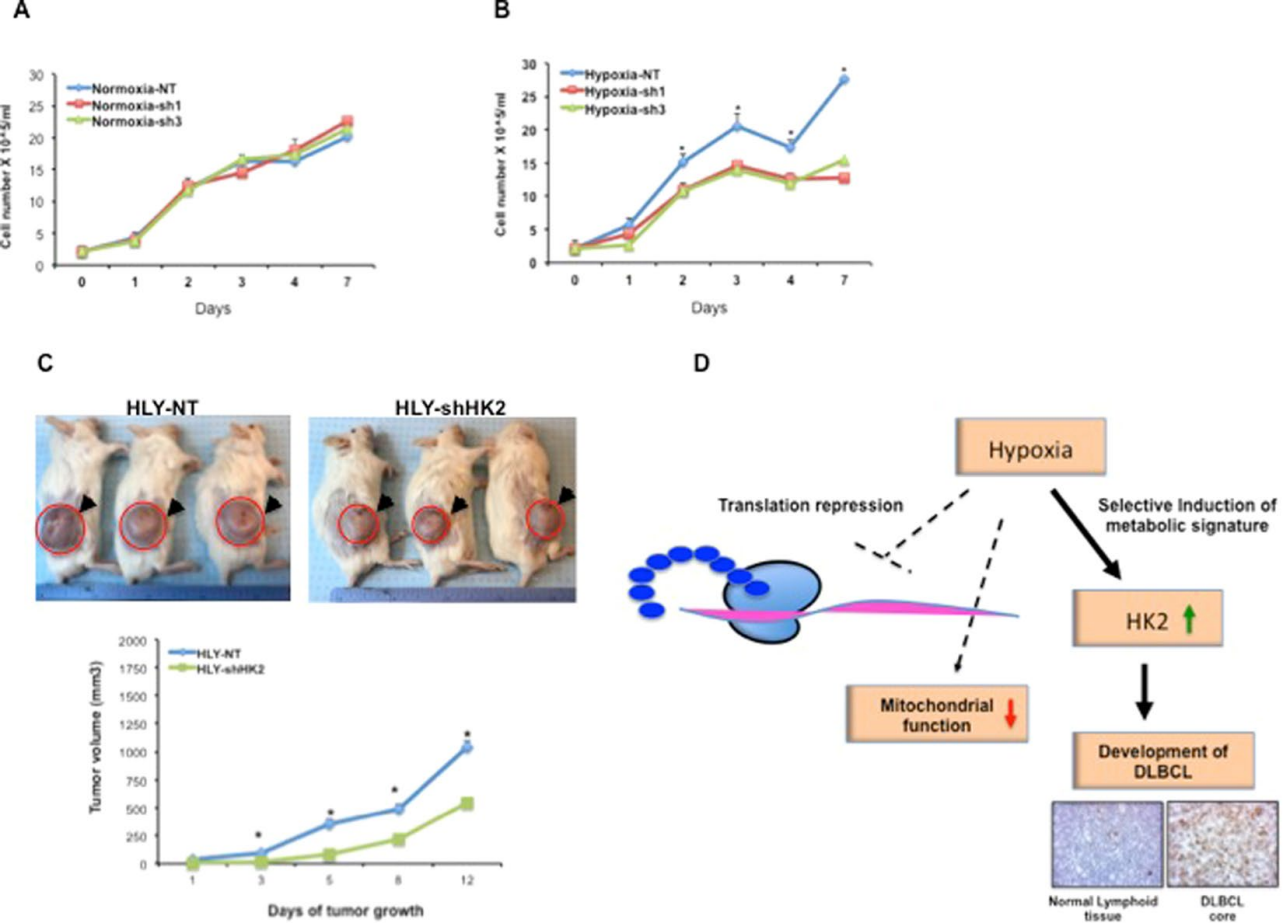
HK2 is required for lymphoma growth. HK2 levels were genetically inhibited using lentiviral particles expressing NT-shRNA and sh-HK2 in HLY cell lines and growth effects were monitored. Total cell counts were performed over a period of one week. (**A**) Normoxia (21% oxygen). (**B**) Hypoxia (1% oxygen). (**C**) The effect of HK2 inhibition on tumorigenesis *in vivo*. HLY cells transduced with NT-shRNA and sh-HK2 lentiviruses were subcutaneously injected into NSG mice and tumor growth was monitored. The top panel shows representative tumor images. Tumor values were calculated from n = 8 animals per group ±SE, p < 0.05. (**D**) Model figure-representing effect of hypoxia on various cellular processes that effectively coordinate to respond to hypoxic stress during DLBCL development.

To further test functional relevance of *HK2* in promoting growth of DLBCL, we genetically inhibited *HK2* signaling. We first checked endogenous levels of *HK2* in DLBCL cell lines (Fig. S8A and B). *HK2* expression was highly induced in lymphoma cell lines compared with a normal B cell line GM02184 and primary B-cells derived from normal lymphoid tissue. To block the level of HK2, lentiviral particles expressing HK2-specific shRNA were used to transduce DLBCL cell lines HLY and SUDHL2. Stably transduced cells with efficient knockdown (Fig. S8C) were used for *in vitro* and *in vivo* studies. Cells expressing non-target (NT) lentivirus were used as control. No change in growth between *HK2*-NT and *HK2*-shRNA expressing cells was observed under standard culture conditions (Fig. 7A). However, the genetic knockdown of *HK2* significantly attenuated growth of HLY-shHK2 cells cultured in hypoxic conditions compared to HLY-NT cells (Fig. 7B). This suggests that *HK2* expression correlates with hypoxic tolerance of lymphoma cells. These results were validated using two different shRNA clones. To exploit the influence of tumor microenvironment in mediating growth effects of *HK2*, we established tumor xenografts in NSG mice using sh-NT and sh-*HK2* expressing DLBCL cells. Cells were inoculated subcutaneously and tumor growth was monitored. Despite a rapid growth in tumor size after a week, knockdown of *HK2* levels was efficient in suppressing tumor growth compared to the control group. The growth of tumors was significantly inhibited by greater than 50% in tumors expressing *HK2*-shRNA compared to tumors bearing NT-shRNA (Fig. 7C). We also performed knockdown of HK2 in another aggressive lymphoma cell line, SUDHL2, to rule out the possibility of a cell line-specific effect. Growth results were similar to what we observed in HLY tumors expressing NT and sh-*HK2* cells. Tumors derived from SUDHL2 cells with HK2 knockdown were about 50% smaller compared to xenograft tumors derived from NT-SUDHL2 cells (Fig. S8D). These studies show a direct role for *HK2* in providing resistance to hypoxic stress and promoting DLBCL tumor growth *in vivo*. In summary, our data support that there is a metabolic coupling of different cellular processes and DLBCL growth during hypoxic stress and the inhibitory influence of metabolic repression on progression of DLBCL is abolished by selective activation of hypoxia target *HK2* (Fig. 7D). Taken together, HK2 expression is a distinct metabolic feature in diffuse B-cell lymphoma and could be an attractive target to exploit for DLBCL therapy.

## Discussion

In this study we first asked whether translation is regulated as an adaptive response to hypoxic stress in lymphoma. Quantitation of protein translation determined that about 50% of translation was inhibited during hypoxia in various DLBCL cell lines. Protein translation is energy intensive. Therefore, suppression of global translation will diminish ATP utilization to protect cell from being energy deprived^19,20^. Indeed our mitochondrial ATP-dependent respiration rates suggested that DLBCL cells were not energy deprived under hypoxic stress.

Our data demonstrates that during hypoxia protein translation was not completely ablated, but was reprogrammed to adapt to hypoxic stress. It has been shown in prostate cancer cell line PC3 that in contrast to global repression under hypoxia, 33 key mRNAs were found to be resistant to repression including glucose transporters^15^. Studies in various other forms of cancers also show that hypoxia stimulates tumor progression by inducing expression of angiogenic, glycolytic, and mitochondrial genes^53–57^. We investigated metabolic gene signatures induced during hypoxic exposure in DLBCL via microarray analysis. RNA enrichment analysis of microarray data linked clinically relevant hypoxia targets to DLBCL phenotype. These selectively induced hypoxia targets included genes of glucose metabolism and mitochondrial function. RT-PCR expression profiling of various DLBCL cell lines demonstrated that expression of key enzymes *GLUT1*, *HK2*, *FAS* and *Cyt-C* was induced during hypoxia in lymphoma cell lines and patient derived malignant B cells. Importantly, expression of these enzymes was not observed in B-cells derived from normal lymphoid tissues. Our transcription data potentially validated that metabolic pathways involved in glucose, lipid and mitochondrial function might mediate DLBCL development.

Based on expression studies, we hypothesized that an increase in mitochondrial function might influence DLBCL growth. However, surprisingly mitochondrial oxygen consumption rate data show that respiration is clamped under hypoxic stress. This was accompanied with a 21% decrease in mitochondrial mass and a decrease in mitochondrial cytochrome c protein expression, which could perhaps account for the reduced oxygen consumption under hypoxic exposure. Interestingly, the effect of OxPhos gene expression observed in nuclear/cytoplasmic fractions was not reflected in mitochondrial function. Respiratory subunits have dual origin and are encoded by both nuclear and mitochondrial genes referred to as nu-OxPhos and mt-OxPhos, for example, cytochrome-c^58-60^. Our data is in line with a recent report demonstrating that nuclear and mitochondrial OxPhos subunits are not coordinately regulated in certain types of cancer^61,62^. It was shown in this study that RNA expression of mitochondrial and nuclear OxPhos subunits was concurrently suppressed compared to normal tissue in the majority of cancer types. In certain types of cancer (breast, bladder, endometrial and lung adenocarcinoma), mitochondrial OxPhos expression was reduced but there was increase in OxPhos subunits in the nuclear fraction. A detailed comparison of mitoribome and cytoribome of OxPhos subunits in DLBCL will provide valuable insights into the role of mitochondria in development of aggressive B cell tumor phenotype. Taken together, our mitochondrial data from DLBCL cell lines predicted a decoupled nuclear and mitochondrial OxPhos regulation during HIF1α activation.

Cells proliferate or undergo cell death depending on the metabolic demand and supply. Therefore, the balance of energy metabolism is very important for cell survival. It has been proposed using two cellular models of hepatocytes and cortical brain cells that a hypo-metabolic state is a prerequisite to surviving hypoxia^63,64^. Genetic knock down of translation factors eef-2, ifg-1 and rpl-6 in C. elegans resulted in decreased oxygen consumption, which was also correlated to cell death resistance after hypoxic exposure^65^. Consistent with these studies, our *in vitro* growth data illustrates that resistance to hypoxic stress is a growth feature associated with translation suppression and reduced oxygen consumption in DLBCL cells during hypoxic stress. It appears that DLBCL cells are able to survive hypoxic stress by depressing their metabolic rate.

To identify functional relevance of changes in *GLUT1*, *HK2* and *Cyt-C* in DLBCL, we performed screening of tissues derived from lymphoma patients and normal individuals, which predicted *HK2* as a critical regulator of lymphoma development. Our data show that availability of *eIF4E1* and *HIF1α* influence effective translation of *HK2* under hypoxia. To further conclusively check if *HK2* alters tumorigenic potential and its relevance to hypoxic environment, we studied the effect of *HK2* knockdown on lymphoma growth *in vitro in* presence of hypoxia and *in vivo* using animal xenograft model, which mimics tumor microenvironment. Our data demonstrate that *HK2* is required for growth-promoting effects in DLBCL. Therefore, targeting of *HK2* translation may be valuable to kill DLBCL cells and prevent disease prognosis.

The glucose analog, 2D-G is the most commonly used pharmacological agent for targeting glucose metabolism^66^. However, drugs that target glycolytic enzymes are not very successful as treatment because they are not specific to tumor cells, and consequently, are associated with high levels of toxicity. Due to these concerns, many recent clinical trials using such inhibitors have been halted^67^. As of yet, there are no clinically effective inhibitors available to target glucose metabolism. Therefore, identification of metabolic partners of *HK2* in large diffuse B-cell lymphoma and targeting them might be an alternative to largely unsuccessful existing drugs for targeting glycolytic enzymes. *HK2* also interacts with mitochondrial proteins such as VDAC and PEA15 and prevents cell death^27^. VDAC targeted therapy can be utilized to disrupt VDAC-HK complexes to improve efficacy of commonly used drugs to inhibit lymphoma growth. Therapies directed to impair *HK2*-mediated metabolic coupling of metabolism and cell survival might have better therapeutic implications.

Currently there is no biomarker available in clinical setting for predicting response to currently used regime for treatment of DLBCL and facilitate stratification of patient population. Although our data does not have direct clinical relevance, *HK2* might have the potential to be a novel metabolic target to predict response to HK2 targeted therapy in DLBCL. Metabolism of tumor cells is reprogrammed for maximum consumption of glucose to provide a carbon source to drive tumor growth^25,68^. It is possible that presence of *HK2* may sensitize DLBCL patients to currently used CHOP-based treatment by depriving dependence of tumors on glucose consumption for its growth and interfering with metabolic needs of tumor.

Collectively, the data presented in this paper demonstrate how metabolic rate depression, specifically repression in global protein translation and mitochondrial function converge to establish a hypoxia protective phenotype of a diffuse large B-cell. This is the first report unraveling the basis of hypoxic control in progression of DLBCL. Furthermore, we have demonstrated that *HK2* contributes to lymphoma development. Importantly, using tissue samples from lymphoma patients, we show that *HK2* expression is strongly associated with development of B cell lymphoma. *HK2* expression may influence prognosis in lymphoma patients. The *HK2* pathway could be one of the causes of tumor relapse in DLBCL subtype of B lymphoma patients. Therefore, specifically targeting *HK2* might yield protection from disease progression. In conclusion, our findings indicate that *HK2* has a potential to be used as a metabolic therapeutic target to stratify the DLBCL patient population to predict clinical response.

## Methods

All methods were performed in accordance with the relevant guidelines and regulations of the University of Maryland Medical School.

### Cell culture, Primary cells and Reagents

SUDHL-2, SUDHL-6, Toledo, GM02184, Karpass 422, U2932, Pfeiffer, OCI-Ly4, OCI-Ly3, Cos-7 cells were obtained from American type culture collection (ATCC). HLY-1 cells were a generous gift from Dr. Lisa Rimsza (University of Arizona, AZ), and Dr. Ari Melnick (Weill Cornell, NY). DLBCL cell lines were grown in RPMI-1640. COS7 and LentiX-293T were grown in DMEM (Cellgro). OCI-Ly4, OCI-Ly3 cells were maintained in IMDM media, which was supplemented with 20% FBS. Single cell suspension of primary cells was obtained from normal and spleen tissue samples collected at the University of Maryland Medical School with institutional guidance and approval. Primary samples (normal lymph nodes and spleens) were provided by UMGCCC (University of Maryland Greenebaum Comprehensive Cancer Center) Pathology. Primary cells were isolated as previously described in^34,69^. Briefly, tissue was chopped into smaller pieces and was then washed with PBS. Cell suspension obtained in RPMI 1640. Lymphocytes were separated from cell suspension using lymphocyte separation media (LSM) at a ratio of 1:1. Cell suspension was layered on LSM and samples were spun at 400 g for 30 min to isolate buffy coat. This was followed by B cell purification using a B cell isolation kit, MACS buffer and magnetic columns (Milynel Biotech) as per manufacturer’s instructions. Primary B cells were cultured in RPMI containing 10% FBS. Cells from lymphoma cell lines and primary tissues were cultured under normoxia (21% oxygen) and hypoxia (1% oxygen) in the presence of 5% CO_2_. Levels of *HK2* were genetically inhibited in HLY and SUDHL-2 cells using shRNA against *HK2*, which was delivered by a lentiviral expression vector. Viral particles and stable cell lines were generated with knockdown of *HK2* using lentiviral-based system as per manufacturer’s details and were selected using 1 µg/ml of puromycin. *HK2* luciferase reporter and eIF4E1 expression plasmid were obtained from Addgene.

### Protein translation

To determine the effect of *HIF1α* induction on protein translation DLBCL cell lines were treated with 100 µM of cobalt chloride (a mimetic for induction of HIF1α). Cells were maintained in normoxia and hypoxia for 48 hrs and were incubated with -[^35^S]-Methionine for one hour. EasyTag^TM^ L-[^35^S]-Methionine, 500 μCi was purchased from Perkin-Elmer. After an hour of ^35^S incorporation cells were harvested, washed with PBS and protein lysates were isolated using 50 µl of lysis buffer. ^35^S-met labeled translation efficiency was measured using a scintillation counter.

### Polysome fractionation

Polysomal fraction was isolated from lymphoma cells lines as previously described^34^. Briefly, lysates were fractionated using sucrose gradient (10–50% sucrose) at 35,000 r.p.m. for 3 hours at 4 °C using Beckman Coulter SW41 rotor. Polysomal lysis buffer consisted of 20 mM Tris-HCL, 100 mM KCL, 5 mM MgCl_2_, 1%Triton and sodium deoxycholate 0.25 g. RNA was isolated using Trizol.

### Western blotting and Antibodies

Western blotting was performed using standard methods. The following primary antibodies were used at a dilution of 1:1000: Anti-glucose transporter Glut-1 (abcam), Hexokinase II (Cell Signaling), Anti-Hif1α (Cayman), Anti-GAPDH (abcam), Anti-eIF4E1 (phospho S209) (abcam), eIF4E1 (Santa Cruz), Anti-Cytochrome C (EPR1327) (abcam), mTOR specific antibodies, rS6k, p-p70s6k, p70s6k and pmTOR were all from Cell Signaling.

### RNA and RT-PCR

Total cytoplasmic RNA, polysomal RNA from sucrose gradients, and RNA from primary samples was extracted using trizol (Ambion). cDNA was synthesized with 1 µg of RNA using high capacity cDNA kit (ABI). For synthesis of cDNA from polysomal RNA, 5 ngs of luciferase RNA was added to each reaction as internal control, which was used to normalize the data^70^. RT-PCR was performed using SYBR green (ABI). Primer sequences are listed in^71^ and below. HK2: F-5′-gag cca cca ctc acc cta ct-3′; R-5′-acc caa agc aca cgc aag tt-3′; GLUT1: F-5′-ggc caa gag tgt gct aaa gaa-3′; R-5′-aca gcg ttg atg cca gac ag-3′; G6PASE: F-5′-act ggc tca acc tcg tct tta-3′, R-5′-cgg aag tgt tgc tgt agt agt ca-3′, LDHA: F-5′-ttg acc tac gtg gct tgg aag-3′, R-5′-ggt aac gga atc ggg ctg aat-3′, SGLT2: F-5′-ctg ttt gca ccc gtg tac ct-3′, R-5′-cct gtc acc gtg taa atc atg g-3′, GLUT4: F-5′tgg gcg gca tga ttt cct c-3′, R-5′gcc agg aca ttg ttg acc ag-3′. PDHB: F-5′-agt ggt ggt gct aga gaa tga-3′, R-5′-tgc agc ttc taa gca gtg gc-3′, OGDH: F-5′ttg gct gga aaa ccc caa aag-3′, R-5′-tgt gct tct acc agg gac tgt-3′; SDHB: F-5′acc ttc cga aga tca tgc aga-3′; R-5′-gtg caa gct aga gtg ttg cct-3′.

### Immunohistochemistry staining

Tissue microarrays (TMAS) were purchased from Biomax (LY800a and LY800b). Tissue samples spotted in the arrays consisted of samples from diffuse large B-cell lymphoma (ABC and GC subtypes), follicular lymphoma, reactive hyperplasia and normal tissues. Each array included 40 DLBCL samples, 5 follicular samples, 15 reactive hyperplasia samples and 20 normal samples. Five follicular lymphoma cases were combined with DLBCL cases spotted in the array for ease of tumor tissue expression data analysis and interpretation. Samples where tissues disc were lost or had high background were excluded from analysis. LY800a and LY800b TMAS were stained with Glut1, HK2 and Cytochrome-C antibodies by Mass histology (MA). Paraffin tissue sections obtained from malignant and normal lymphoid tissue were stained with HIF1α antibody. A hematologist at the University of Maryland evaluated immuno-histochemical staining independently. Tissue cores that were lost during the staining procedure or tissue demonstrating unclear staining were excluded from analysis.

### Microarray Analysis

Illumina Human HT-12 v4 expression microarray gene chip (Illumina) containing 48,000 RefSeq transcripts was used to generate microarray data. Total RNA quantity and quality was tested using the Agilent Bioanalyzer RNA 6000 Chip (Agilent, Santa Clara, CA). Five hundred ng total RNA was labeled according to the manufacturer’s instructions using the Illumina array, Total PrepTM RNA amplification kit (Illumina, San Diego, CA). A total of 750 ng biotinylated aRNA was hybridized to the Illumina HumanHT-12 v4 Expression BeadChip overnight. Following post hybridization rinses, arrays were incubated with streptavidin-conjugated Cy3, and scanned at a resolution of 0.53 μm using an Illumina iScan scanner. Hybridization intensity data were extracted from the scanned images using Illumina BeadStudio GenomeStudio software, V2011.1.Genes were considered altered if Z > 1.5 and p value ≤ 0.02 was obtained. Pathway analysis was performed to identify genes that were differentially regulated in hypoxia compared to normoxia. Microarray was first filtered by signal detection p value or detection score. For each pair, the z-test p value <=0.05 with fdr <=0.30 and z-ratio absolute value do not less than 1.5 were selected, with ANOVA F-test p value less than 0.05 and detection selection within the cutoff were selected as significant gene set for each pairwise comparisons. The significant gene expression change as z-ratio from the whole microarray gene expression change were used as input to perform on pathway gene sets, to specifically perform mitochondrial functional level gene expression change effects.

### Measurement of oxygen consumption rate and mitochondrial staining

Mitochondrial oxygen consumption rate was measured in DLBCL cell lines (HLY, Karpass 422, Farage and Toledo) that were identified to have an OxPhos phenotype based on RNA expression studies. Cells were incubated overnight in normoxia (21% oxygen, 95% air, 5% CO_2_), or in an oxygen-regulated incubator 3%O_2_, 92% N_2_, 5% CO_2_ (hypoxia) for OCR experiments. Oxygen consumption measurements were performed using an XF24 Extracellular Flux Analyzer and Wave software (Agilent Technologies, Santa Clara, CA, USA). DLBCL cells were cultured in RPMI and 5 × 10^5^ cells in a volume of 200 μL were seeded on XF24 V7 plates to measure OCR using Seahorse media with 10 mM glucose, 2 mM glutamine, 1 mM pyruvate. Seahorse XF24 V7 plates were coated with the cell adhesive Cell-Tak (BD, Bioscience, Bedford, MA, USA) prior to cell plating as per manufacturer’s instructions. The plates were spun at 200 rpm (with deceleration set to “off”) and incubated at 37 °C for 10 min for complete cell attachment. Assay plates were then placed in a CO_2_-free incubator at 37 °C. After 30 minutes, an additional 475 uL of assay media was added to bring the volume up to 675 uL. Seahorse assay measurement cycles were set to 3 min mix, 2 min wait, and 2 min measure. Three measurements of basal respiration were taken prior to any drug addition. Drug injection ports sequentially added increasing concentrations of the uncoupler 2,4-dinitrophenol (DNP, 25 µM added per injection) to stimulate maximal respiration. An additional 10 mM pyruvate was also added during the first injection to stimulate the production of the Complex I substrate NADH. The Complex III inhibitor Antimycin A (1 µM) was added last and any remaining respiration subsequent to antimycin A addition was considered non-mitochondrial respiration. ATP-linked respiration, defined as the difference between basal respiration and respiration after the addition of the ATP synthase inhibitor oligomycin (0.5 µg/mL), was measured in separate wells, with oligomycin titrated instead of DNP. Measurements were taken for a total of 60 minutes. Glycolysis was measured in Seahorse media containing 1 mM glutamine. Extracellular acidification rate (ECAR) was measured to estimate the rate of glycolytic lactic acid production in Seahorse media containing 1 mM glutamine. Glycolysis was defined as the difference between the ECAR after the addition of glucose (10 mM) and after the injection of 2-deoxyglucose (25 mM). Data was analyzed using the software SIGMA PLOT 12.0. Assessment of changes in mitochondrial mass during hypoxic exposure was done using the fluorescent probe MitoTracker Green (MTG) by flow cytometry, as suggested in published studies^72^. To detect mitochondrial related changes in DLBCL cells during hypoxia, we also performed live imaging of cells using MTG. Immunofluorescence staining of mitochondria was done using antibodies to detect mitochondrial proteins Tom20 and cytochrome-c. Mitophagy was measured using CCCP (carbonyl cyanide m-chlorophenyl hydrazine) and HCQ (hydroxy-chloroquine). Cells were treated overnight with CCCP (25 µM) and then next day for 3 hours with 25 µM HCQ before samples were read for Mito Tracker Green fluorescence by flow cytometry.

### Flow Cytometry growth studies

1 × 10^6^ cells per 2 ml were seeded in 6 well plates and maintained in 21% and 1% oxygen for 24 hours to measure cell growth. The numbers of live and dead cells were distinguished using Live and dead assay kit (Molecular Probes) and FACS Canto-II as per manufacturer’s instructions. 0.25 × 10^6^ cells were stained as per manufacturer’s recommendation and green and red fluorescence were measured at ~495/515 nm and ~495/635 nm excitation/emission spectra, respectively. For growth arrest assays 4.8 × 10^5^ cells were cultured at 21 and 1% oxygen and pelleted after 48 hrs for fixing and staining for cell cycle distribution analysis. The pellet was washed 2× with cold PBS and cells were centrifuged at 400 rcf for 5 minutes. 75% of cold ethanol was added to the pellets and cells were fixed overnight at 4 °C. The next day, cells were centrifuged at 400 rcf for 5 min and stained with propidium iodide (PI) at room temperature for 30 minutes in dark. The stain consisted 25 µg/ml of PI, 1% Triton and 1 mg of RNAase. Cell cycle was analyzed by FACS Canto-II.

### Luciferase promoter assay

HKII promoter construct^47^ and pcDNA3-HA-eIF4E1 expression plasmid were purchased from Addgene. To perform luciferase reporter assays COS7 cells were cultured under 1% hypoxia in the presence or absence of *HK2* promoter plasmid and co-transfected with other plasmids including *HIF1α*, and *eIF4E1*.

### Animal experiments and cell line xenografts

Animal procedures were performed by the translational core facility at the University of Maryland. The animal protocol was approved by the Institutional Animal Care and Use Committee of the University of Maryland. NSG mice were subcutaneously injected in flank with DLBCL cell lines (HLY-1 and SUDHL2) transduced with NT-shRNA or Sh-HK2 lentivirus. 1 × 10^6^ cells were resuspended in 200 µl of PBS and mixed with matrigel and the mixture was subcutaneously injected into the left and right dorsal flanks of 5–7 weeks old NSG male mice. Upon establishment, tumors growth was measured three times a week until tumor volume reached 2 mm^3^. Animals were euthanized and tumors were collected and frozen at −80 °C for further expression analysis.

### Statistical analyses

All statistical analyses were performed using pairwise comparisons and Student t-test and were expressed as ±(SE) standard error of mean. Correspondence analysis was performed to correlate expression of *HK2*, *GLUT1*, and *Cyt-C* with DLBCL phenotype. Correspondence analysis (CA) graph was obtained using CA package of the statistical language R.

### Data availability

Microarray data generated in this study is deposited in NCBI database with the GEO accession number GSE104212 and NCBI tracking number 18660401.

## Electronic supplementary material

Supplementary Information
