# Author Correction: Role of hypoxia in Diffuse Large B-cell Lymphoma: Metabolic repression and selective translation of HK2 facilitates development of DLBCL

**DOI:** 10.1038/s41598-018-25251-9

**Published:** 2018-05-03

**Authors:** Kavita Bhalla, Sausan Jaber, Nanaji Nahid M., Karen Underwood, Afshin Beheshti, Ari Landon, Binny Bhandary, Paul Bastian, Andrew M. Evens, John Haley, Brian Polster, Ronald B. Gartenhaus

**Affiliations:** 1 Marlene and Stewart Greenebaum Comprehensive Cancer Center, University of Maryland, Department of Medicine, Baltimore, MD 21201 USA; 2 University of Maryland, Department of Biochemistry and Molecular Biology, Baltimore, MD 21201 USA; 3 Veterans Administration Medical Center, Baltimore, MD 21201 USA; 4 University of Maryland, Flow Cytometry Core, Greenebaum Comprehensive Cancer Center, Baltimore, MD 21201 USA; 5 Tufts Medical Center, Boston, MA 02111 USA; 6 Yale School of Medicine, Yale University, New Haven, CT 06520 USA; 7 National Institute on Aging, National Institutes of Health, Baltimore, MD 21224 USA; 8 Department of Pathology, Stony Brook Medicine, Stony Brook, NY 11794-8691 USA

Correction to: *Scientific Reports* 10.1038/s41598-018-19182-8, published online 15 January 2018

The original version of this Article contained a typographical error in the spelling of the author Paul Bastian, which was incorrectly given as Paul Bastain.

In addition, the Acknowledgements section in this Article was incorrect:

“We thank Dr. Shambhu Bhat for performing correspondence analysis in this study and also for critical reading of the paper. We thank Dr. Girnun for providing HIF1α construct and valuable suggestions for our study. We thank Dr. Elin Lehrmann and Dr. Kevin Becker for assistance with microarray data. We are grateful to Drs. Tony Passaniti and Michele Vitolo for critical reading of the manuscript. The research in R.B.G laboratory was supported in part by a Merit Review Award from the Department of Veterans Affairs and RO1-10019169 and RO1-10018832 from the NIH.”

now reads:

“We thank Dr. Girnun for providing HIF1α construct and valuable suggestions for our study. We thank Dr. Shambhu Bhat for performing correspondence analysis in this study and also for critical reading of the paper. We thank Dr. Elin Lehrmann and Dr. Kevin G. Becker (National Institute on Aging, National Institutes of Health Intramural Research Program) for undertaking microarray experiments, data analysis, and GEO data submission. We are grateful to Dr. Tony Passaniti and Dr. Michele Vitolo for critical reading of the manuscript. The research in R.B.G laboratory was supported in part by a Merit Review Award from the Department of Veterans Affairs, RO1-10019169 and RO1-10018832 from the NIH. This research was also supported in part by the Intramural research Program of the NIH, National institute on Aging.”

These errors have now been corrected in the PDF and HTML versions of the Article.

