# Supplementary material for: Role of hypoxia in Diffuse Large B-cell Lymphoma: Metabolic repression and selective translation of HK2 facilitates development of DLBCL
Source: Sci Rep. 2018 Jan 15;8:744. doi: 10.1038/s41598-018-19182-8 (PMC5768748; doi:10.1038/s41598-018-19182-8)
Supplement: Supplementary file 1 — Supplementary Information [file 41598_2018_19182_MOESM1_ESM.pdf]

Title page

Role of hypoxia in Diffuse Large B-cell Lymphoma: Metabolic repression and selective translation of HK2 facilitates development of DLBCL

Kavita Bhalla[1,*], Susan Jaber[2], Nanaji Nahid M[3], Karen Underwood[4], Afshin Beheshti[5], Ari Landon[6], Binny Bhandary[1], Paul Bastain[7], Andrew M. Evens[5], John Haley[8], Brian Polster[2], and Ronald B. Gartenhaus[1,3*]

[1]Marlene and Stewart Greenebaum Comprehensive Cancer Center, University of Maryland, Department of Medicine, Baltimore MD 21201, USA; [2]University of Maryland, Department of Biochemistry and Molecular Biology, Baltimore MD 21201, USA; [3]Veterans Administration Medical Center, Baltimore, MD 21201, USA; [4]University of Maryland, Flow Cytometry Core, Greenebaum Comprehensive Cancer Center, Baltimore MD 21201, USA; [5]Tufts Medical Center, Boston, MA, 02111; [6]Yale School of Medicine, Yale University, New Haven, CT, 06520; [7]National Institute on Aging, National Institutes of Health, Baltimore, MD 21224, [8]Department of Pathology, Stony Brook Medicine, Stony Brook, NY 11794-8691, USA.

Supplemental Material

Figure S1. Western blot analysis of mTORC targets in HLY and SUDHL2. HLY and SUDHL2 cells were grown for different time points at 1 % oxygen tension. Cells grown under normoxia were used as controls for the time points indicated in the figure. Protein lysates were obtained and expression levels of pMTOR, pRibosomalS6K, and p70S6K were determined using antibodies from Cell Signaling. GAPDH was used as an internal control for sample loading.

Figure S2. Microarray analysis of DLBCL cell lines HLY and SUDHL2. Co-correlation analysis of microarray data generated showed RNA enrichment of clinically relevant hypoxia metabolic targets in our dataset. Each column represents a sample for **(A)** HLY and **(B)** SUDHL2 cell lines that were cultured under normoxia and hypoxia, n=3. HLY and SUDHL2 cells were cultured in normal culture conditions and 1% hypoxia and microarray analysis was performed using Illumina gene chip. **(C)** Gene enrichment analysis of HIF1α targets in the SUDHL2 dataset identified increased expression of glucose transporters SLC2A1 (GLUT1) and HK2 in hypoxia samples compared to those derived from normoxic cells. Top panel: heat map, bottom panel: fold change, *$p<0.05$, n=3, $\pm$SD. **(D)** List of ribosomal regulated genes.

Figure S3. Ingenuity Pathway (IPA) toxicity analyses. IPA toxicity analysis of microarray data in **(A)** HLY cell line, and **(C)** SUDHL2 cell line. Pathway analysis was filtered for pathways pertaining to mitochondria and hypoxia. Top mitochondrial pathways regulated under hypoxia are listed based on level of significance that are regulated under hypoxia are listed. X-axis is the –log of a p value and Y-axis represents different mitochondrial pathways regulated in DLBCL cell lines. (B and D) A detailed list of mitochondria-related genes that are regulated in respective mitochondrial pathways under hypoxia in HYL and SUDHL2 cell lines as determined by IPA toxicity analysis. (B, HLY) and (D, SUDHL2).

Figure S4. Expression of HIF1α targets in normal B-cells derived from tonsils. Expression of HIF1α targets was determined by RT-PCR analysis in primary B-cells derived from normal tonsils. There was no change in expression of glucose transporters in normal B-cells obtained from processing of tonsil tissue. However, the HIF1α target VEGF was significantly induced under short-term hypoxic exposure of B cells compared to cells cultured under normoxia, $p<0.05$, n=3, ±SD.

Figure S5. Measurement of ATP dependent OCR and immunofluorescence staining of mitochondrial markers in DLBCL cells. **(A)** ATP synthesis-dependent oxygen consumption rates of cells treated with hypoxia were unchanged relative to normoxia in DLBCL cell lines K422, HLY and Toledo. **(B)** HLY and Farage cells were also stained with MitoTracker green (MTG) to detect changes in mitochondrial mass. **(C and D)** Immunofluorescence staining of mitochondrial protein TOM20 and Cytochrome-C in DLBCL cells exposed to normoxia or hypoxia. There was intense staining of TOM20 and Cty-C in normoxic cells compared to cells cultured in 1% hypoxia.

Figure S6. Growth characterization of DLBCL cells under hypoxic stress. **(A)** N=normoxia; H=hypoxia, $p<0.05$, Flow cytometry data of cell cycle distribution are represented as a graph. For visualization purposes, each histogram is divided into three quadrants, each representing a G1/S/G2-M phase, as indicated in the figure. The percentage of cells accumulated in G2/M phase was significantly different from cells cultured in normoxia, $p<0.05$. Results are expressed as n=3 ±sd for A, B and C respectively. **(B and C)** Expression of markers indicative of cell cycle progression in HLY and SUDHL2 cells. Markers of mitotic induction (*CDC25B* and *CDC2*) were downregulated in SUDHL2 cells under hypoxic stress. In contrast, expression of *CDC25B* and *CDC2*, *CDC25A* were up-regulated in HLY cells when grown in 1% oxygen compared to normoxic control.

Figure S7. The presence of *eIF4E1* selectively stimulates expression of *HK2* under hypoxia. **(A)** Detection of *peIF4E1* and *eIF4E1* at indicated time points in HLY and SUDHL2 cells exposed to hypoxic stress. **(B and C)** Polysomal fractions were isolated from HLY and SUDHL2 cells cultured under normoxia and hypoxia and analyzed for *eIF4E1* and *HK2* levels by western blot analysis. **(D)** HLY cells were transfected with *eIF4E1* and selected with G418. Stably transfected cells were then subjected to normal culture conditions or hypoxic stress for 48 hrs. Protein lysates were obtained and expression of HK2 was determined in the presence or absence of *eIF4E1*. **(E)** Luciferase assay using *HK2* promoter in COS7 cells to determine the effect of *eIF4E1* and *HIF1*α on *HK2* promoter activity. COS7 cells transfected with *eIF4E1*, *HIF1*α or both were incubated in 21% oxygen (normoxia) or 1 % oxygen (hypoxia) for 24 hrs and luciferase activity was measured using Promega dual activity luciferase kit. Renilla was used as an internal control.

Figure S8. Effect of *HK2* in promoting tumor growth. **(A and B)** RNA and protein expression of endogenous *HK2* levels in non-malignant B-cell line GM02184 (GM), primary B-cells derived form normal tonsil tissue (T1, T2) and DLBCL cell lines HLY, SUDHL2 and SUDHL6. **(C)** Knockdown of HK2 levels in HLY using lentivirus (Origene). Cells expressed non-target shRNA (NT), shRNA HK2 (Clones: sh5VA, sh5VB, sh5VC and sh5VD). shRNA clones sh5VA and sh5VB with efficient knock down were used for *in vitro* and *in vivo* growth experiments. **(D)** Xenograft tumors were established in NSG mice (n=5 per group, NT and shHK2) using SUDHL2 cells expressing NT or shHK2 lentivirus. Growth of tumors was monitored for approximately 2 weeks and tumor volume was measured.

Figure S1

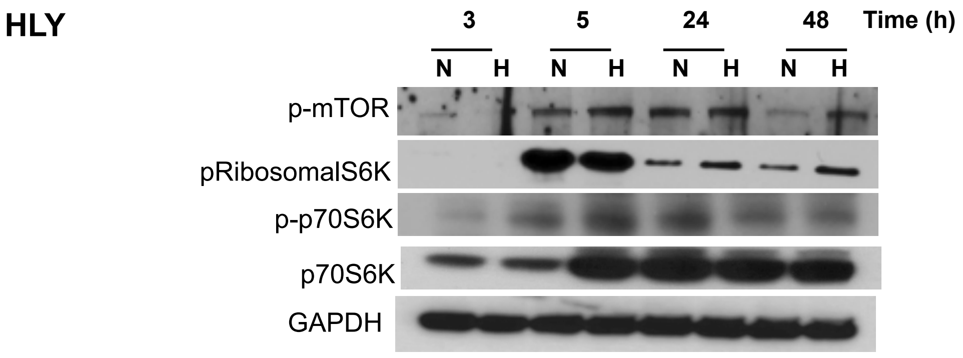
HLY
3
5
24
48
Time (h)
N
H
N
H
N
H
N
H
p-mTOR
pRibosomalS6K
p-p70S6K
p70S6K
GAPDH

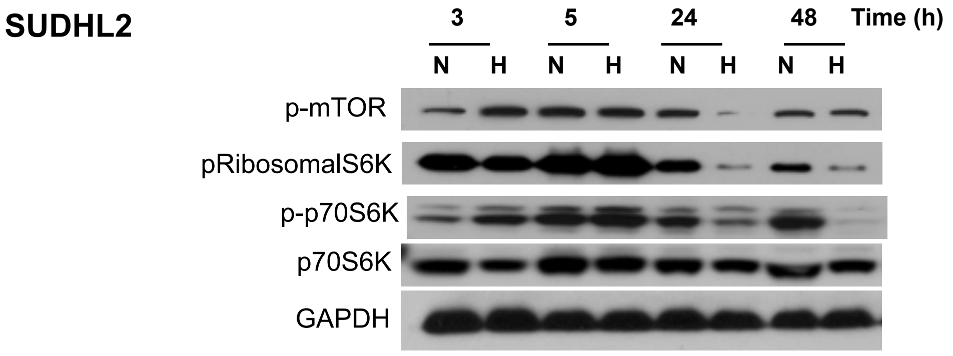
SUDHL2
3
5
24
48
Time (h)
N
H
N
H
N
H
N
H
p-mTOR
pRibosomalS6K
p-p70S6K
p70S6K
GAPDH

Figure S2

A

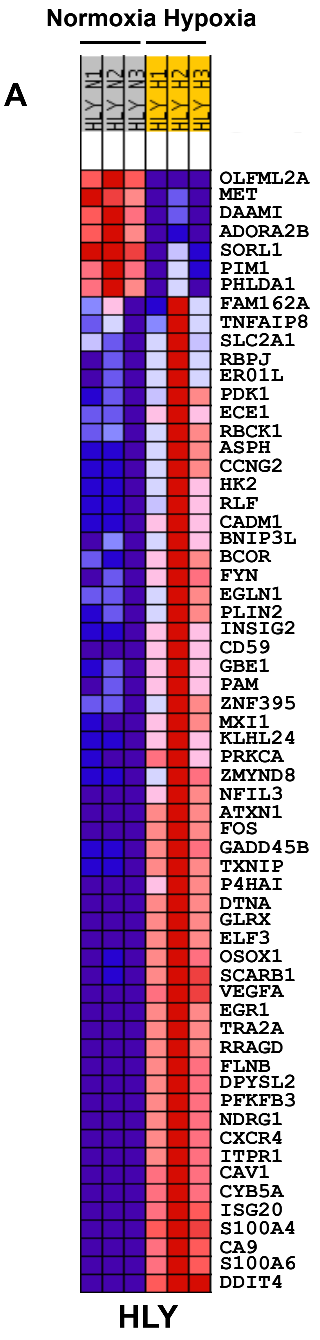

B

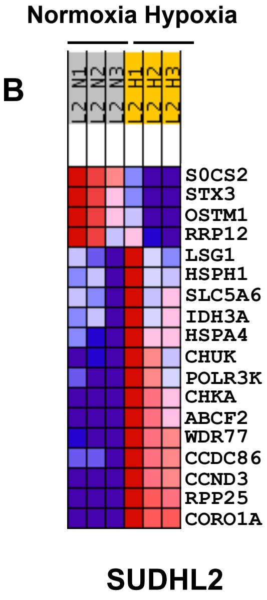

D

TargetID	HLY1	SUDHL2	Acc	Name
MRPL41	-1.7	2.06	Q8IXM3	39S ribosomal protein L41, mitochondrial
MRPL43	-1.34	1.78	M0R051	39S ribosomal protein L43, mitochondrial
MRPS2	-1.05	1.6	Q96Q47	Mitochondrial ribosomal protein S2
RPF2	1.08	-1.03	Q9H7B2	Ribosome production factor 2 homolog
RPL15	1.62	-1.13	E7EQV9	Ribosomal protein L15
RPL22	1.22	-1.04	K7EP65	60S ribosomal protein L22
RPL26L1	-2.53	2.85	E5RIT6	60S ribosomal protein L26-like 1
RPL28	2.04	-1.15	H0YMF4	60S ribosomal protein L28
RPL36A	2.18	-1.64	R4GN19	60S ribosomal protein L36a
RPS23	3.54	-2.62	D6RIX0	40S ribosomal protein S23
RPS27L	1.49	-1.31	C9JLI6	40S ribosomal protein S27

C

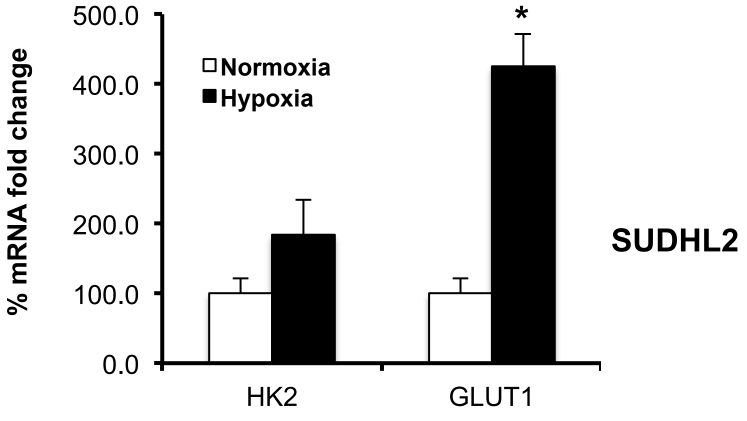

Figure S3

A **HLY**

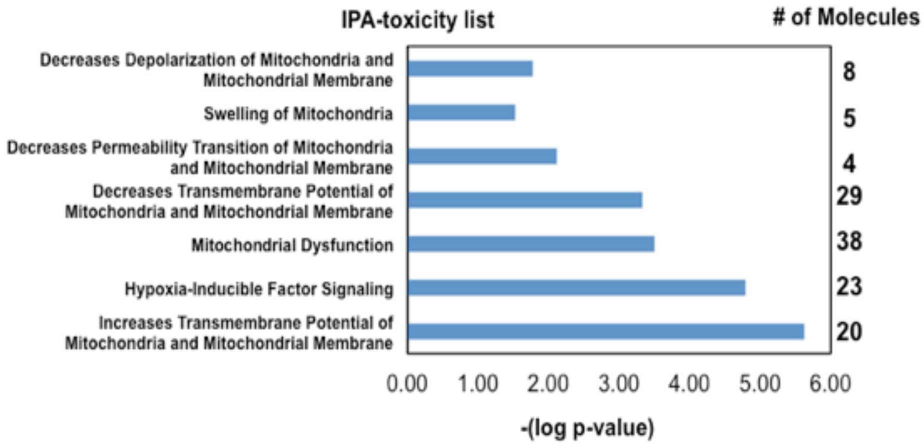

C **SUDHL2**

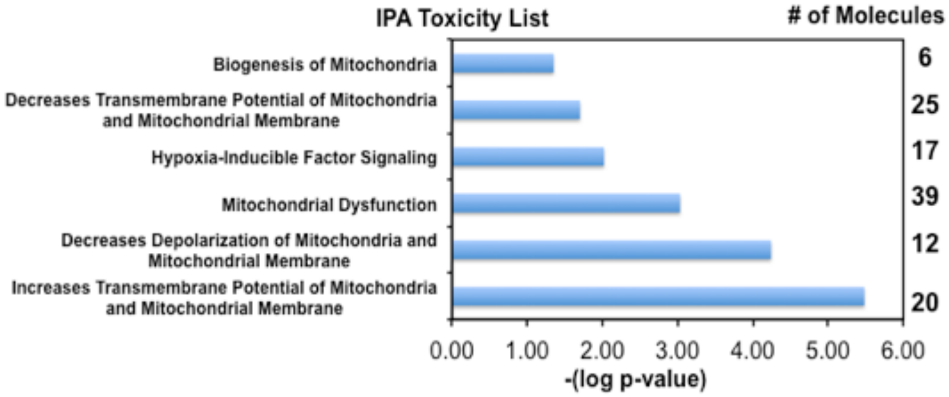

B **HLY**

Pathways	Molecules
Increase in transmembrane potential of MM	PTPN6,YWHAE,CASP3,RAC1,IL6,FAS,BCL2,HSPA4, SOD2,CD40,PIM1,HGF,CAT,CYCS,CFLAR,CD27, CASP7,PPARGC1A,LGALS1,TCL1A
Hypoxia inducible factor signaling	TP53,PIK3CA,EIF2B4,NQO1,MDM2,HIF1A,NOS3, UBE2L6,EIF2S2,EP300,VEGFA,PTK2,SHC1,UBE2J1, EIF1,UBE2H,CREB1,HIF1AN,EIF2B5,UBE2C,ATM,PR KCB,UBE2I
Mitochondrial dysfunction	FURIN,NDUFA7,ACO2,TRAK1,NCSTN,NDUFB8, PSEN2,ATP5S,SDHAP1,CYB5R3,BCL2,NDUFB9, SOD2,ATPAF1,GPX4,ACO1,OGDH,AIFM1,NDUFA8, SDHA,UCP2,CASP3,COX7A2L,NDUFB1,MAPK12, GPX7,GSR,FIS1,NDUFA6,UQCR10,CAT,UQCRC2, COX5A,APH1B,SDHD,CYCS,CYB5A,BACE2,COX15
Decrease in transmembrane potential of MM	CDKN2A,AATF,PYCARD,MUC1,MAPK13,FAS,DFFA, BCL2,MYC,SHC1,ZMAT3,SOD2,BID,IFI6,TP53,GZMA, UCP2,CASP3,AGA,PAWR,TCF3,ATPIF1,CAMP,SLC25 A14,CASP2,PRKCD,CAT,RTN4,TNF,HSPB1
Decrease permeability transition of MM	BTG2,OGDH,HSPA5,BCL2
Swelling of mitochondria	TP53,TNF,FAS,BCL2,AIFM1
Decrease depolarization of mitochondria and Mitochondrial membrane	CAT,CDKN1A,PPIA,FAS,CSTB,MCL1,PPARGC1A, BCL2

D **SUDHL2**

Pathways	Molecules
Increase in transmembrane potential of MM	PTPN6,CASP3,ABL1,RAC1,FAS,BCL2,HSPA4,PRDX, SOD2,CD40,PHB,PIM1,HGF,CAT,CFLAR,CD27,CASP7, PPARGC1A,LGALS1,TCL1A
Decrease depolarization of MM	PAK1,CAT,CDKN1A,PPIA,PLIN3,MAP2K1,ATP2A2,FAS, CSTB,MCL1,PPARGC1A,BCL2
Mitochondrial dysfunction	FURIN,ATP5G1,ATP5D,NDUFA7,ACO2,TRAK1,NCST N,NDUFB8,PSEN2,ATP5S,BCL2,NDUFB9,SOD2,ATPA F1,NDUFB6,GPX4,AIFM1,NDUFA8,ATP5G2,NDUFAF, UCP2,CASP3,GLRX2,NDUFB1,MAPK12,GPX7,GSR, FIS1,PRDX3,CAT,UQCRC2,COX5A,APH1B,SDHD, BACE2,CYB5A,NDUFS3,COX15
Hypoxia inducible factor signaling	EIF2B4,CSNK1D,PLCG1,MDM2,HIF1A,NOS3,UBE2L6, EIF2S2,EP300,UBE2D4,PTK2,JUN,UBE2J1,HIF1AN, EIF2B5,PRKCB,UBE2I
Decrease in transmembrane potential of MM	GZMA,CDKN2A,UCP2,CASP3,AATF,PYCARD,MUC1,A GA,MAPK13,TCF3,NFKB1,FAS,BCL2,FADD,DIABLO, SOD2,SLC25A14,PRKCD,CAT,RTN4,BCL2L2,BID,IFI6, TNF,HSPB1
Biogenesis of Mitochondria	PRDX3,GNAS,TFAM,SHARPIN,MAN2A1,PPARGC1A

Figure S4

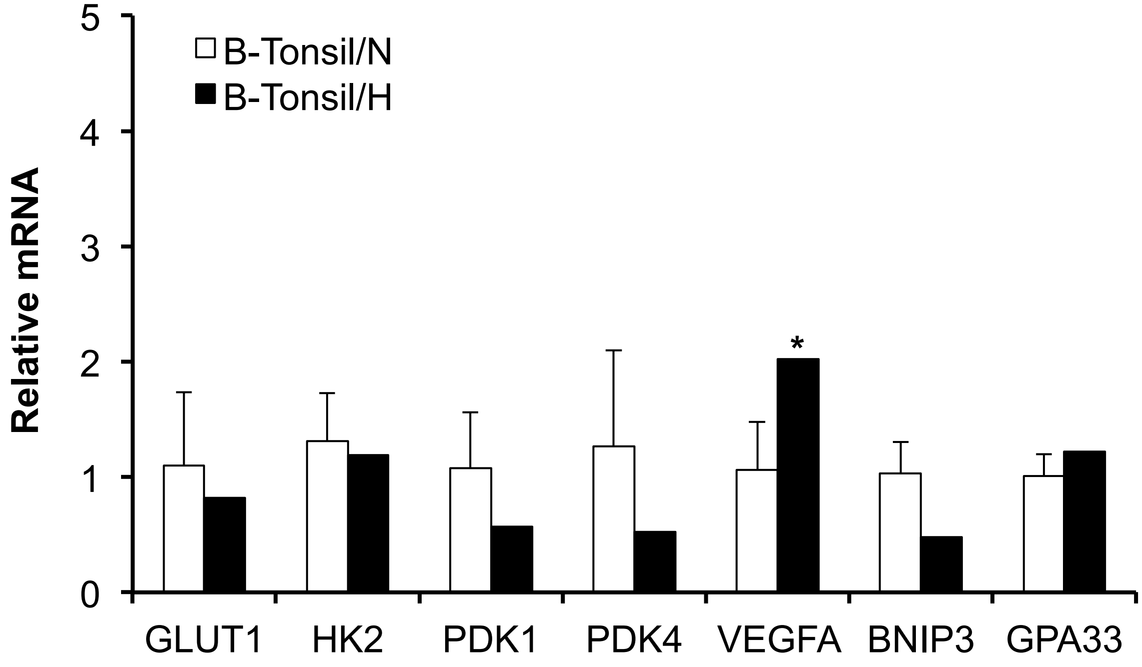

B-Tonsil/N
B-Tonsil/H
Relative mRNA
5
4
3
2
1
0
*
GLUT1
HK2
PDK1
PDK4
VEGFA
BNIP3
GPA33

Figure S5

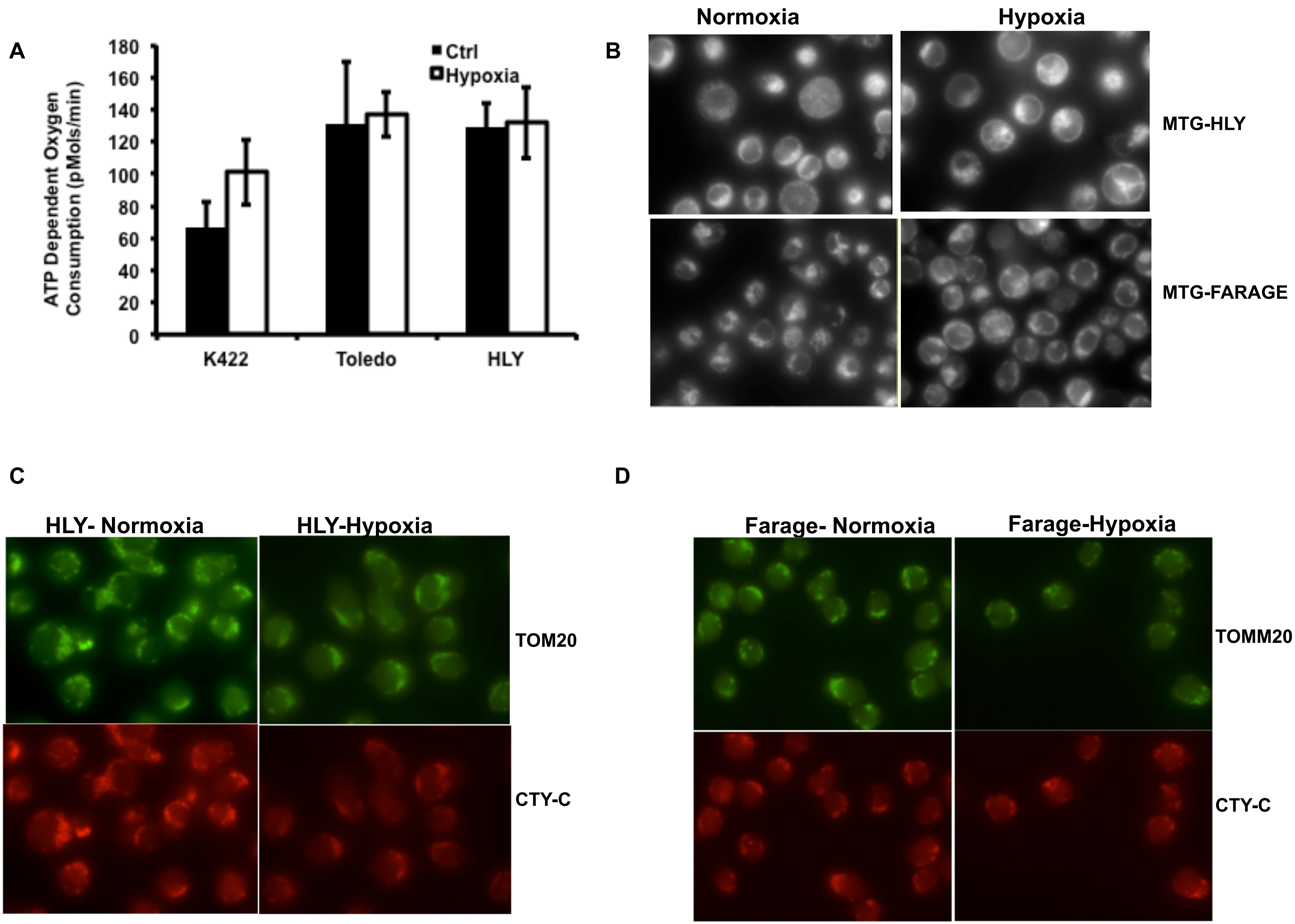
A
180
160
140
120
100
80
60
40
20
0
ATP Dependent Oxygen Consumption (pMols/min)
Ctrl
Hypoxia
K422
Toledo
HLY
B
Normoxia
Hypoxia
MTG-HLY
MTG-FARAGE
C
HLY- Normoxia
HLY-Hypoxia
TOM20
CTY-C
D
Farage- Normoxia
Farage-Hypoxia
TOMM20
CTY-C

Figure S6

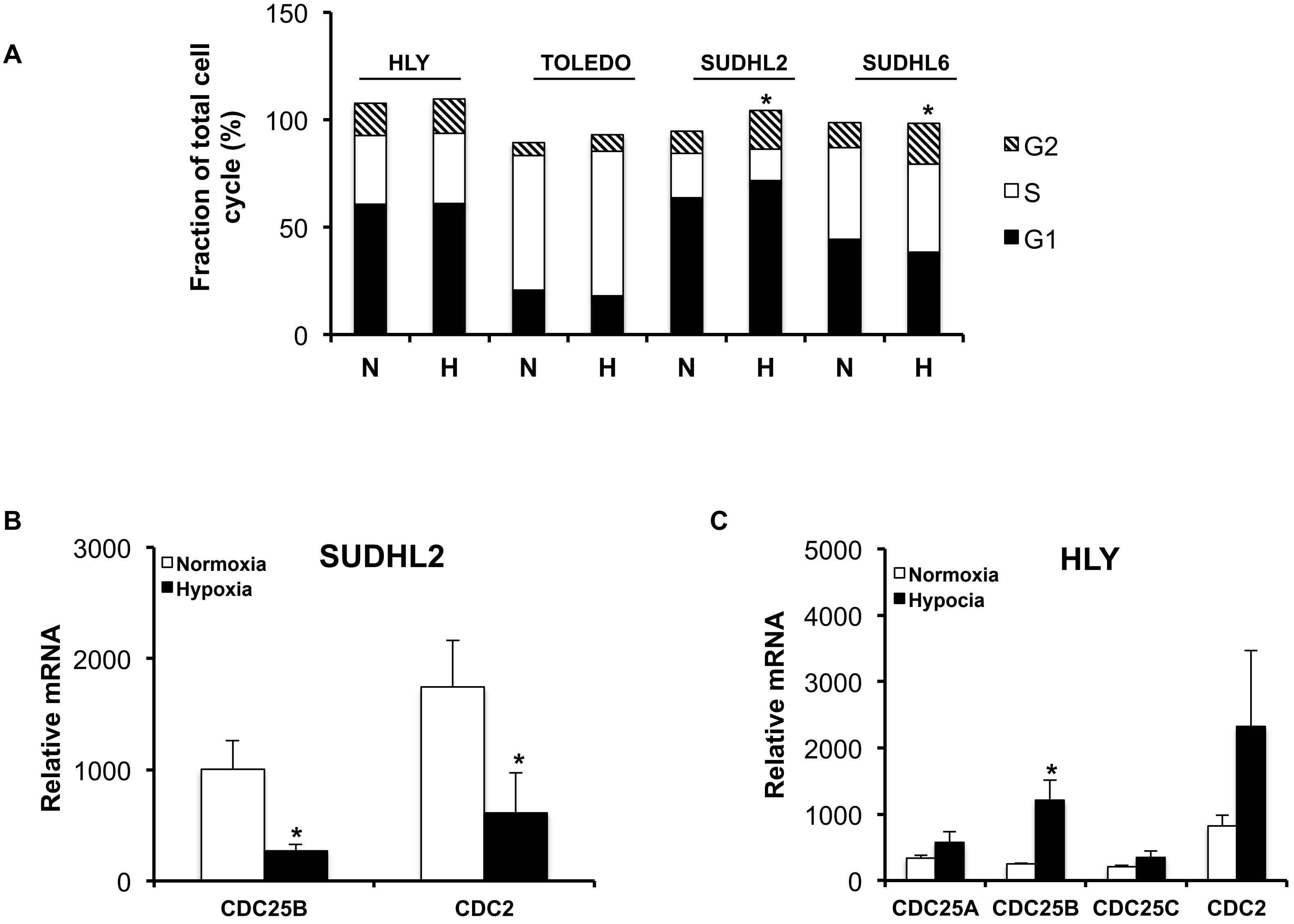
A
150
100
50
0
Fraction of total cell cycle (%)
HLY
TOLEDO
SUDHL2
SUDHL6
*
*
G2
S
G1
N
H
N
H
N
H
N
H
B
3000
2000
1000
0
Normoxia
Hypoxia
SUDHL2
Relative mRNA
*
*
CDC25B
CDC2
C
5000
4000
3000
2000
1000
0
Normoxia
Hypocia
HLY
Relative mRNA
*
CDC25A
CDC25B
CDC25C
CDC2

Figure S7

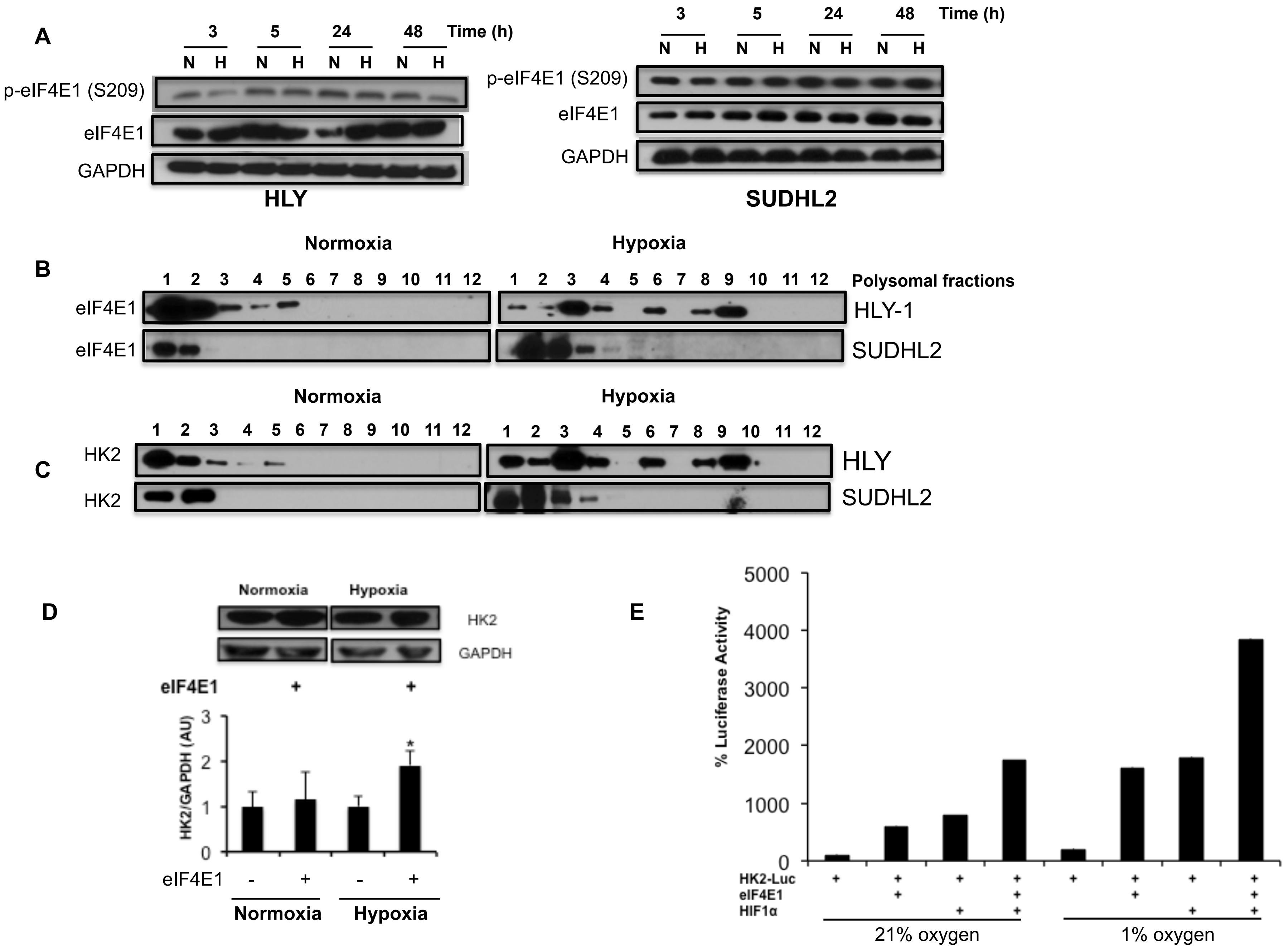
A
3
5
24
48
Time (h)
N H N H N H N H
p-eIF4E1 (S209)
eIF4E1
GAPDH
HLY
SUDHL2
B
Normoxia
Hypoxia
1 2 3 4 5 6 7 8 9 10 11 12
Polysomal fractions
eIF4E1
HLY-1
SUDHL2
C
HK2
HLY
SUDHL2
D
Normoxia
Hypoxia
HK2
GAPDH
eIF4E1
+
HK2/GAPDH (AU)
0
1
2
3
*
eIF4E1 - + - +
E
% Luciferase Activity
0
1000
2000
3000
4000
5000
HK2-Luc
eIF4E1
HIF1α
21% oxygen
1% oxygen

Figure S8

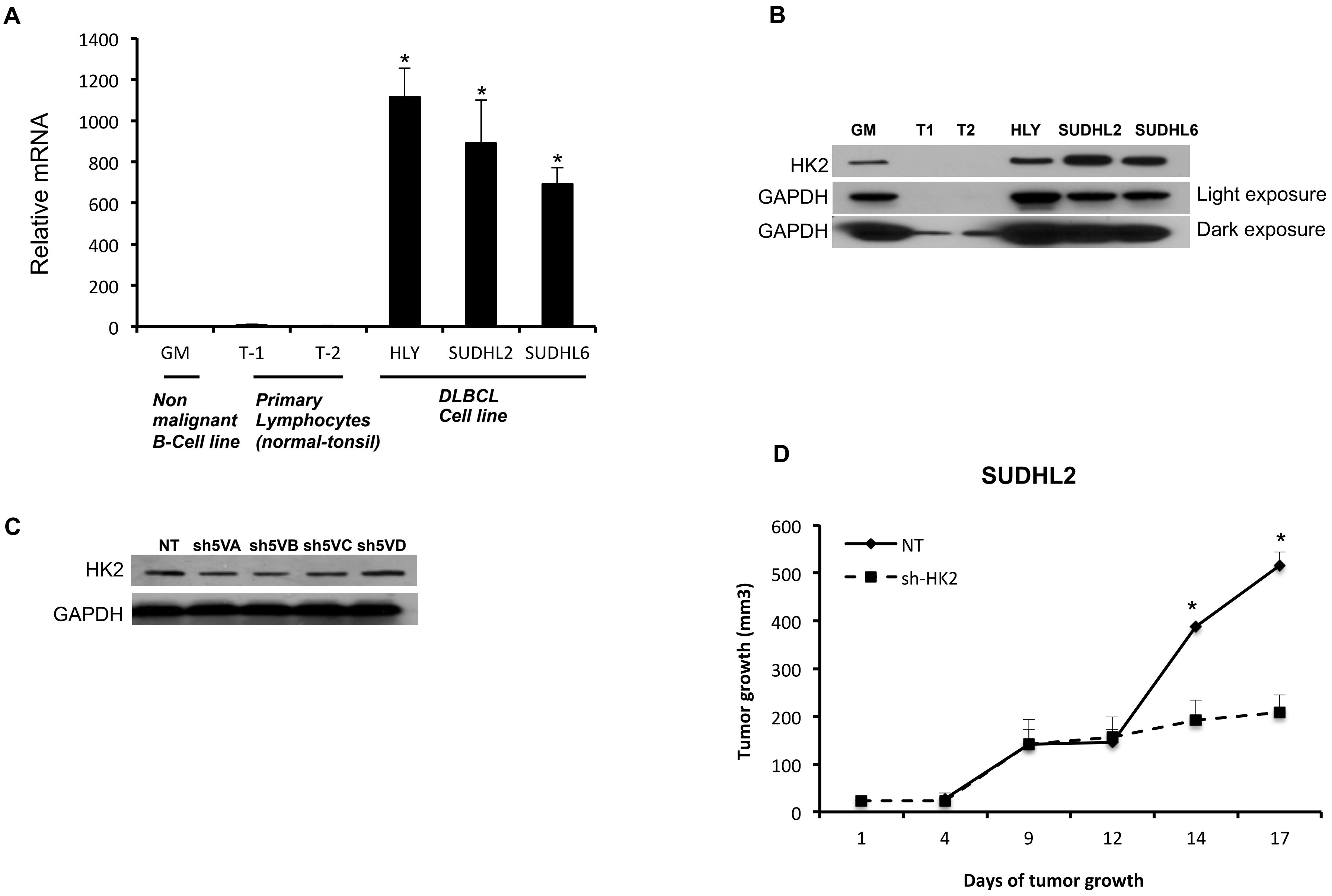

A
Relative mRNA
1400
1200
1000
800
600
400
200
0
*
GM
T-1
T-2
HLY
SUDHL2
SUDHL6
Non malignant B-Cell line
Primary Lymphocytes (normal-tonsil)
DLBCL Cell line
B
GM
T1
T2
HLY
SUDHL2
SUDHL6
HK2
GAPDH
Light exposure
GAPDH
Dark exposure
C
NT
sh5VA
sh5VB
sh5VC
sh5VD
HK2
GAPDH
D
SUDHL2
NT
sh-HK2
Tumor growth (mm3)
600
500
400
300
200
100
0
1
4
9
12
14
17
Days of tumor growth
